# Beyond Steady-State: An Integrated Framework Unveils BPAP as the Highest-Risk Bisphenol in a Dynamic River System

**DOI:** 10.3390/toxics14050448

**Published:** 2026-05-20

**Authors:** Zheng Zhang, Lulu Zhang, Jingru Zhang, Lingyun Yu, Yujun Tong, Qiusen Huang, Yueping Zhu, Wenyu Xie, Dongpo Liu

**Affiliations:** 1 Laboratory of Risk Assessment and Control of New Pollutants, Guangdong Provincial Academy of Environmental Sciences, Guangzhou 510045, China; 16616646626@163.com (Z.Z.); lulu5013@126.com (L.Z.); zhangjrgucas@163.com (J.Z.); ylygdaes@163.com (L.Y.); ryantongyujun@163.com (Y.T.); hqssunmoon@163.com (Q.H.); 2 School of Environmental Science and Engineering, Guangdong University of Petrochemical Technology, Maoming 525000, China; gdmmzyp@163.com (Y.Z.); gdmmxwy@163.com (W.X.)

**Keywords:** bisphenol compounds, Environmental Condition Index (ECI), Toxicological Priority Index (ToxPi), sediment–water interface processes, dynamic risk, watershed management

## Abstract

The paradigm for managing emerging contaminants is shifting from static concentration control toward dynamic risk forecasting. However, this transition is hindered by the lack of mechanistic models that can link nonlinear environmental processes to holistic risk prioritisation. Here, we present an integrated modelling framework that resolves driver collinearity, integrates multimedia risks, and apportions pollution sources. The framework combines three novel components: an Environmental Condition Index (ECI) to quantify synergistic environmental influences on contaminant release, an extended dual-media Toxicological Priority Index (ToxPi) for holistic risk integration, and an enhanced Positive Matrix Factorisation model (PMF-DMC) for spatially resolved source attribution. Applied to a complex watershed in the Pearl River Basin, the framework revealed a critical risk priority reversal: bisphenol AP (BPAP) emerged as the top-priority control pollutant, contrary to concentration-centric assessments that identified bisphenol A (BPA). This reversal underscores the inherent limitations of concentration-centric regulation and demonstrates the necessity of adopting dynamic, process-informed frameworks for managing emerging contaminants. The framework’s transferable design establishes it as a predictive and adaptive tool for risk governance, offering a methodological advance for ecological modelling in non-stationary environments.

## 1. Introduction

The management of emerging contaminants is evolving from a paradigm of static concentration control towards one of dynamic risk forecasting [1,2,3]. This critical transition, however, is impeded by a persistent gap between the capabilities of contemporary ecological models and the complex, non-stationary realities of multi-stressor watersheds. While models for contaminant fate and ecological risk assessment provide foundational tools, their frequent reliance on steady-state assumptions fails to capture how episodic events, such as floods, fundamentally alter source and sink relationships and risk hierarchies [4,5]. This inadequacy is particularly acute for organic contaminants like bisphenol analogues (BPs), where regulatory restrictions on bisphenol A (BPA) have increased the use of substitutes (e.g., BPF, BPS, BPAP), raising concerns over “regrettable substitution” [4].

The core challenge lies in the inability of conventional frameworks to mechanistically link nonlinear environmental processes to holistic, multi-dimensional risk prioritisation [6,7]. This manifests in two interconnected methodological bottlenecks. First, predictive modelling of key processes, such as contaminant partitioning, is often compromised by strong collinearity between co-varying environmental drivers (e.g., dissolved organic carbon and temperature), obscuring their individual versus synergistic effects [8,9]. Second, risk assessment frameworks heavily reliant on scalar metrics like the risk quotient (RQ) inadequately account for multi-dimensional attributes (e.g., differential toxicity, cross-media bioavailability), leading to the systematic deprioritisation of low-concentration but high-potency substances [10,11]. This “steady-state blindness” is especially consequential for emerging compounds like BPAP with elevated toxicological potency, resulting in systematic risk underestimation [12]. Furthermore, while tools like the Toxicological Priority Index (ToxPi) offer multi-criteria integration, their application to dual-media (water–sediment) systems at the watershed scale for spatially differentiated management remains underexplored [13,14].

These limitations have direct management implications in composite functional watersheds. The Foshan reach of the Pearl River Basin exemplifies such a complex system, characterised by high population density, significant industrial and agricultural activity, and multiple drinking water intakes [15,16,17]. Here, the steady-state paradigm is demonstrably inadequate. It fails to elucidate the “process black box” of how co-varying factors interact nonlinearly to influence contaminant release from sediments, a potential latent “risk reservoir” during hydrological perturbations [18]. Critically, these gaps reinforce each other: unresolved process uncertainty undermines exposure estimates, while inadequate prioritisation frameworks cannot capture how episodic events may invert established risk hierarchies [19].

To bridge these critical knowledge gaps, we propose and validate an integrated modelling framework designed to transform risk assessment from a static inventory into a dynamic, predictive tool. The framework achieves this by synergistically combining components into a causal process–risk–source chain. Specifically, we (1) quantify synergistic environmental control on contaminant release via a novel composite index (ECI) to resolve driver collinearity and diagnose the “when and why” of sediment activation; (2) integrate multi-dimensional risks across water and sediment media via an extended ToxPi to identify “which contaminant poses the greatest integrated threat” under the dynamic exposure scenarios diagnosed by the ECI; and (3) apportion pollution sources in a spatially resolved manner via an enhanced PMF-DMC model to answer “where the risk originates”. It is this formal integration of process diagnosis, holistic risk integration, and source attribution that allows the framework to uncover emergent phenomena, such as the risk priority reversal among bisphenol analogues, which are systematically overlooked by steady-state paradigms that treat these components in isolation. By applying this framework to the complex Foshan reach of the Pearl River Basin, we aim to elucidate the mechanisms behind these reversals, thereby testing its capacity to uncover latent threats.

## 2. Materials and Methods

### 2.1. Study Area and Data Collection

The study was conducted in the Foshan section of the Pearl River Basin, encompassing the Xijiang and Beijiang tributaries. This is a typical region within the Guangdong–Hong Kong–Macao Greater Bay Area, characterised by highly overlapping industrial, agricultural, and water resource functions [20]. The Xijiang flows through agricultural and light industrial areas, while the Beijiang traverses highly industrialised districts (e.g., Sanshui, Nanhai) with concentrated plastics, chemical, and electronics manufacturing [21,22]. This spatial juxtaposition of distinct anthropogenic influences within a single hydrological unit provides an ideal setting to investigate emerging contaminant dynamics.

**Figure 1:**
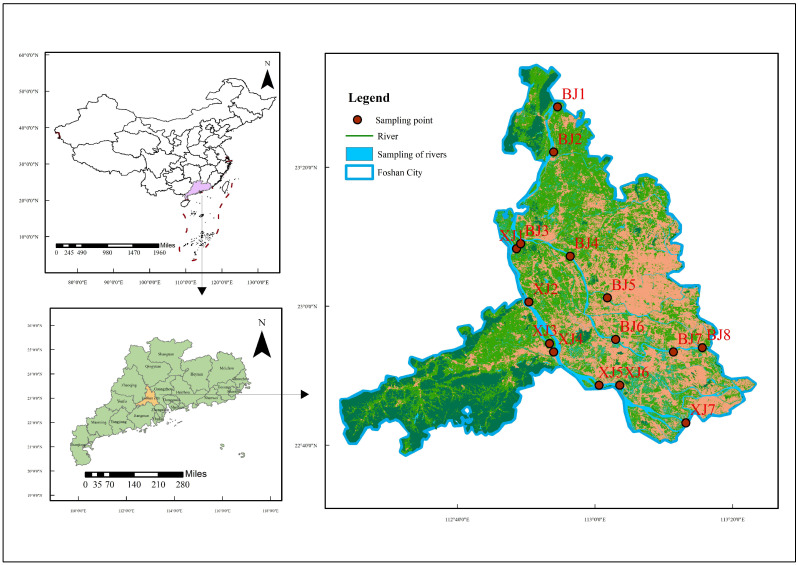
Map of the study area and locations of sampling sections. Arrows show logical zoom-in progression across three maps.

Fifteen representative monitoring sections were established (Figure 1, Table S1): seven on the Xijiang (XJ1–XJ7) and eight on the Beijiang (BJ1–BJ8). Sections included watershed inlets (background), outlets (control), national and provincial assessment points, and areas adjacent to key drinking water intakes, enabling characterisation of spatial pollution patterns. A total of 30 water–sediment sample pairs were theoretically collected (15 sites × 2 campaigns). However, due to site-specific constraints, 20 valid pairs were used for analysis. During the late flood season, eight sites (XJ5, XJ7, BJ2, BJ4, BJ6, BJ7, BJ8, BJ3) yielded insufficient sediment mass because of bed scouring under high-flow conditions, and their sediment samples were excluded. For two sites in the early flood season (XJ4 and BJ1), the water–sediment partition coefficients for key target compounds fell outside acceptable QA/QC limits, and these pairs were excluded to prevent analytical bias.

Sampling captured seasonal hydrological influences. Campaigns were conducted during stable conditions in the early flood season (Xijiang: June; Beijiang: July 2024) and after extreme events in the late flood season (August 2024 for both rivers) [23,24], with no rainfall 48 h prior (Text S1). Water samples were collected using pre-cleaned 1L amber glass bottles. In situ parameters (temperature, pH, DO, conductivity) were measured with a YSI ProDSS multi-parameter analyser (YSI Inc., Yellow Springs, OH, USA) (Table S2). Rainfall data were sourced from the ECMWF ERA5-Land dataset (European Centre for Medium-Range Weather Forecasts, Reading, UK. Available online: https://www.ecmwf.int/en/forecasts/datasets (accessed on 3 January 2026), Table S3).

Water samples were acidified (pH < 2 with HCl), sealed, transported at 4 °C in the dark, and processed within a week. Surface sediments (0–5 cm) were collected with a stainless steel grab sampler, sieved (2 mm), and stored in amber glass bottles, and frozen at −20 °C until analysis.

### 2.2. Chemical Analysis and Quality Control

The target compounds included 12 bisphenols (BPs) (Table S4). However, due to the absence of key aquatic toxicity data for BPAS and BPAG, and the non-detection of BPB and BPZ in sediment samples, subsequent risk assessment and ToxPi analysis were restricted to the remaining 10 BPs with complete toxicity profiles. To determine whether this exclusion affects the overall risk ranking, a supplementary analysis was conducted using available monitoring data. For BPAS and BPAG, even under the most conservative assumption that their toxicities are equivalent to that of BPA, their contributions to total risk would be negligible. Specifically, their mean concentrations in water (1.41 ng/L and 0.03 ng/L, respectively) are 2–3 orders of magnitude lower than that of BPA (71.8 ng/L). Regarding BPB and BPZ, which were not detected in any sediment sample and exhibited extremely low detection frequencies in water (13% and 7%, respectively), the maximum calculated Hazard Quotients (HQs), derived from their respective oral reference doses (RfDs) (Table S8) and maximum detected concentrations (0.03 ng/L and 0.09 ng/L), are on the order of 10^−6^ to 10^−7^. These values are more than six orders of magnitude below the risk threshold (HQ = 1) and far below the risk levels of BPA and BPAP. Therefore, the exclusion of these four compounds does not alter the priority ranking of the remaining 10 bisphenol compounds.

For sample preparation, 1 L water samples were filtered through a 0.7 μm glass fibre filter, the pH adjusted to 5–7, and liquid–liquid extraction performed with 50 mL of acetonitrile after adding internal standards and 5 g of NaCl. Sediment samples (approximately 2.0 g dry weight) were extracted using an accelerated solvent extractor (ASE 350, Dionex, Sunnyvale, CA, USA) with a 1:1 (*v*/*v*) mixture of dichloromethane: acetone at 100 °C and 1500 psi, followed by cleanup on a composite SPE cartridge. Full procedural details for both matrices are provided in Texts S2.1 and S2.2. For instrumental analysis, all BP extracts were analysed using a liquid chromatography–tandem mass spectrometry system (LC-MS/MS; LC-30AD UHPLC, Shimadzu, Kyoto, Japan; coupled to a QTRAP^®^ 5500, AB Sciex, Framingham, MA, USA) with an electrospray ionisation source operating in negative ion mode. Separation was performed on a Waters ACQUITY UPLC^®^ HSS C18 column (2.1 × 100 mm, 1.8 μm, Waters Corporation, Milford, MA, USA) at 40 °C. The mobile phase consisted of 2 mM ammonium acetate in water (A) and acetonitrile (B). The injection volume was 2 μL. The gradient programme and multiple reaction monitoring (MRM) parameters are detailed in Text S2.3.

A rigorous QA/QC programme was implemented to ensure data integrity, as described in Text S2.4. Quantification was performed using the internal standard method (e.g., BPA-d_16_) with a multi-point calibration curve for each target BP. Method detection limits (MDLs), determined at a signal-to-noise ratio (S/N) of 3, were 0.05 ng L^−1^ for water and 0.025 ng g^−1^ dry weight (dw) for sediments (Table S4). Limits of quantification (LOQs) were defined as the lowest calibration standard meeting S/N ≥ 10 and acceptable accuracy (80–120%). For concentrations between the MDL and LOQ, we assigned a value of 0.5 × LOQ, with an estimated uncertainty of ±50%. A full suite of QA/QC samples—including laboratory blanks, matrix blanks, matrix spikes (MS), and matrix spike duplicates (MSDs)—was analysed after every 20 environmental samples. No target BPs were detected in any blank sample. The mean recovery of the 12 BPs in matrix-spiked samples was 93.1% ± 7.93% (*n* = 6 per matrix), and surrogate recoveries in field samples ranged from 56.3% ± 12.1% to 113% ± 22.3% (n = 3–5 per compound), all within established QA/QC acceptance criteria (40–130%). Instrumental stability was monitored by analysing a calibration verification standard every ten samples; response deviations for all target compounds remained within ±20%. All reported concentrations were consistently within acceptable ranges.

### 2.3. Development and Integration of the Modelling Framework

The analytical core of this study is the development and integration of three complementary mathematical models designed to diagnose environmental processes, integrate multi-dimensional risks, and apportion pollution sources within a dynamic watershed system.

#### 2.3.1. Process Diagnostic Model: Development and Validation of the Environmental Condition Index (ECI)

The sediment–water distribution coefficient ($K_{d}$ L/g) was first calculated to quantify the partitioning behaviour of BPs between phases (Equation (1), Table S5). This served as the primary response variable for model development.

(1) $$K_{d}\;=\frac{C_{s}}{C_{w}}$$

where K_d_ is the sediment–water distribution coefficient (L/g), and C_s_ and C_w_ are the concentrations of BPs in sediment (dry weight, ng/g) and water (ng/L), respectively.

The daily average pollution load flux (F, kg/d) was subsequently calculated to quantify the downstream transport load of BPs, providing a complementary metric to assess the environmental consequence of the partitioning dynamics predicted by the ECI model (Table S6):

(2) $$F\;=\;C\;\times \;Q\;\times \;8.64\;\times \;10^{-5}$$

where C is the measured average concentration at the monitoring section (ng/L), and Q is the discharge at the section during sampling (m^3^/s). The factor 8.64 × 10^−5^ is used for unit conversion to obtain the daily load flux F in kg/d.

Initial attempts to predict logK_d_ using standard multiple linear regression with dissolved organic carbon (DOC), logK_ow_, and water temperature (T) as predictors were unsuccessful. The model exhibited severe multicollinearity due to the near-perfect co-variation in DOC and T between the early and late flood seasons (r = 0.98, *p* < 0.001). This resulted in unreliable coefficient estimates and low explanatory power (R^2^ = 0.14), preventing the quantitative separation of their individual and synergistic effects on contaminant partitioning. To resolve this collinearity and capture the dynamic interplay of these drivers [25,26], a novel composite metric, the ECI, was formulated. The ECI is defined as the weighted sum of standardised DOC and T values:

(3) $$\mathrm{ECI}\;=\;w_{1}\times \;\frac{\mathrm{DOC}}{{\mathrm{DOC}}_{\mathrm{std}}}+w_{2}\times \;\frac{T}{T_{\mathrm{std}}}$$

where DOC_std_ = 2.63 mg/L and T_std_ = 28.4 °C [27,28] are reference values for normalisation. The weighting scheme (W_1_ = 0.3 for DOC, W_2_ = 0.7 for T) was informed by prior studies indicating temperature is the primary thermodynamic driver for desorption [29], with DOC playing a secondary, conditional role as a complexing agent. Sensitivity analysis (Figure S1) confirmed that the core model outcome (a negative coefficient c for ECI in Equation (4)) was robust across a plausible range of weight ratios. The ECI thus quantitatively represents the synergistic condition, concurrently elevated T and high DOC, which promotes contaminant transfer from sediments [30,31].

A theoretically constrained partitioning model was then established using the ECI:

(4) $$\log K_{d}\;=\;b\;\times \log K_{\mathrm{ow}}\;+\;c\;\times \;\mathrm{ECI}\;+\;d\;$$

Model coefficients were constrained a priori: a positive b (hydrophobicity effect) and a negative c (ECI-driven desorption effect). Regression on valid paired data yielded [32,33]: b = +0.42, c = −1.85, d = +4.52 (R^2^ = 0.68). To quantify parameter uncertainty, bootstrap resampling with 10,000 iterations provided 95% confidence intervals: b [0.32, 0.52], c [−2.26, −1.44], d [3.84, 5.19] (Figure S2). The strong negative c coefficient quantitatively confirms that transient environmental forcing (ECI), rather than intrinsic hydrophobicity, governs phase partitioning during hydrologically active periods. DOC concentrations were estimated using a region-specific model (DOC = 0.42 × COD_Mn_ + 0.35) refined with district-level land use data to account for spatial heterogeneity ([34,35,36,37] and Table S7).

The weighting coefficients for DOC (W_1_) and T (W_1_) within the ECI were selected via a decision-support matrix that simultaneously evaluated four key metrics: (i) model fit, as indicated by the R^2^ of the regression between the ECI and logK_d_; (ii) the magnitude of the release effect (|c|); (iii) the stability of the resultant risk rankings; and (iv) the balance between the two input variables. As illustrated in Figure S1, a temperature weight of 0.7 provides the best compromise across all four criteria. Thus, the chosen weights are data-driven, derived from this calibration process, ensuring that the ECI serves as a robust, transparent, and process-informed diagnostic tool. The full results of the sensitivity analysis are presented in Figure S1.

#### 2.3.2. ToxPi Model Construction and Comprehensive Priority Ranking

To generate the necessary input data for the risk integration model, conventional ecological and health risk assessments were first conducted. The ecological risk quotient (RQ) was calculated as follows:

(5) $${\mathrm{RQ}}_{i}\;=\;\frac{{\mathrm{MEC}}_{i}}{{\mathrm{PNEC}}_{i}}$$

(6) $$RQ_{T}=\sum_{i}{\mathrm{RQ}}_{i}$$

(7) $${\mathrm{PNEC}}_{i}=\frac{{\mathrm{LC}}_{50}\;\mathrm{or}\;{\mathrm{EC}}_{50}\;\mathrm{or}\;\mathrm{NOEC}}{\mathrm{AF}}$$

where RQ_i_ is the ecological risk quotient for bisphenol analogue i; MEC_i_ is the measured environmental concentration used for exposure assessment of compound i, μg/L; PNEC_i_ is the predicted no-effect concentration for compound (Table S8), μg/L; and RQ_T_ is the total ecological risk quotient for all detected BPs [38,39,40]. PNEC values were primarily obtained from the authoritative NORMAN Ecotoxicology Database and the published literature. For compounds missing from the database (e.g., BPAG, 4-TOP), the US EPA ECOSAR (Ecological Structure Activity Relationships) software (https://www.epa.gov/tsca-screening-tools/ecological-structure-activity-relationships-program-ecosar-updates-ecosar-v111 (accessed on 3 January 2026)) was used to predict acute toxicity data (${\mathrm{LC}}_{50}$/${\mathrm{EC}}_{50}$) for algae, daphnia, and fish. The most sensitive species’ toxicity endpoint was selected, and an assessment factor of 1000 (for acute data) was applied to derive the PNEC value. Risk classification criteria were: RQ < 0.01 (no risk), 0.01 ≤ RQ < 0.1 (low risk), 0.1 ≤ RQ < 1.0 (medium risk), RQ ≥ 1.0 (high risk).

To assess non-carcinogenic health risks pertinent to the watershed’s drinking water source function, the hazard quotient (HQ) was employed:

(8) $${\mathrm{HQ}}_{i}=\frac{{\mathrm{CDI}}_{i}}{{\mathrm{RFD}}_{i}}$$

(9) $${\mathrm{CDI}}_{i}=\frac{{M\times C}_{\mathrm{wi}}}{A}$$

(10) $${\mathrm{HQ}}_{T}=\sum_{i}{HQ}_{i}$$

where HQ_i_ is the non-carcinogenic risk quotient for BP i via drinking water ingestion; CDI_i_ is the estimated daily intake per unit body weight for BP i via drinking water, ng/(kg·d); RfD_i_ is the reference dose for BP i, ng/(kg·d, Table S8); M is the daily average drinking water intake, L/d; $C_{wi}$ is the concentration of BP i in the drinking water environment, ng/L; A is the body weight, kg. Body weight (A) and daily average drinking water intake (M) are model parameters. Due to individual variability, using fixed parameters may lead to underestimation or overestimation of health risk levels. To rigorously characterise this uncertainty and compensate for limitations in sample size regarding parameter variability, this study adopted the probabilistic framework from Yu et al. (2025) [41], employing Monte Carlo simulation (10,000 iterations) for uncertainty analysis of the HQ. This approach propagates the uncertainty in input parameters (e.g., body weight, daily water intake, concentration variability) to generate a probability distribution of the HQ output, providing a more robust risk characterisation than deterministic point estimates. The resulting cumulative distribution functions for HQ across population groups (children/adults) allowed us to report risk probabilities (e.g., the likelihood that HQ exceeds a threshold). An HQ value > 1 indicates that BPs i may pose a non-carcinogenic risk to human health; HQ ≤ 1 suggests the risk is small or negligible. This study referenced data from [42,43,44], setting the daily average drinking water intake for children and adults at 1.25 and 1.95 L/d, respectively, and body weights at 16.68 and 57.03 kg, respectively. It should be noted that the HQ values presented in this study are based on measured concentrations in raw river water, collected before any drinking water treatment. As water treatment processes (e.g., coagulation, sedimentation, filtration, disinfection) can substantially reduce the concentrations of organic contaminants, the reported HQs likely overestimate the actual human exposure risk. Therefore, these HQs should be interpreted as a conservative, screening-level indicator of raw-water hazard, rather than a precise estimate of exposure risk from treated tap water.

To overcome the limitations of single-metric risk rankings, an extended Toxicological Priority Index (ToxPi) model was implemented for holistic, multi-criteria integration [14]. The framework incorporated six standardised dimensions across water and sediment media: (1) Detection Frequency (exposure prevalence); (2) Mean Concentration (exposure intensity); (3) Predicted No-Effect Concentration (inherent ecological toxicity); (4) Ecological Risk Quotient (current ecological risk); (5) Relative Potency Factor (inherent health toxicity); and (6) Health Risk Quotient (current health risk). Although some of these indicators are mathematically related (e.g., RQ = MEC/PNEC; HQ = CDI/RfD), each captures a distinct dimension of the risk profile: exposure magnitude, ecological hazard, human health hazard, and their integrated outcomes. This allows them to collectively provide a more comprehensive and robust prioritisation than any single indicator alone. Reflecting the watershed’s function as a critical drinking water source within the Guangdong–Hong Kong–Macao Greater Bay Area, health-related indicators (dimensions 5 and 6) were assigned double the weight (W = 2) of other indicators (W = 1) [45]. This weighting aligns with the high-priority management goal of protecting drinking water sources, as reflected in regional water quality protection plans. The specific calculation formula is as follows:

(11) $${\mathrm{ToxPi}}_{\mathrm{score}}=w_{1}\frac{{\mathrm{DF}}_{\max}-{\mathrm{DF}}_{\min}}{\mathrm{DF}-{\mathrm{DF}}_{\min}}+w_{2}\frac{{\mathrm{MC}}_{\max}-{\mathrm{MC}}_{\min}}{\mathrm{MC}-{\mathrm{MC}}_{\min}}+w_{3}\frac{{\mathrm{RQ}}_{\max}{-\mathrm{RQ}}_{\min}}{\mathrm{RQ}-{\mathrm{RQ}}_{\min}}+w_{4}\frac{{\mathrm{PNEC}}_{\max}-{\mathrm{PNEC}}_{\min}}{\mathrm{PNEC}-{\mathrm{PNEC}}_{\min}}\;\;+w_{5}\frac{{\mathrm{HQ}}_{\max}-{\mathrm{HQ}}_{\min}}{\mathrm{HQ}-{\mathrm{HQ}}_{\min}}{+w}_{6}\frac{{\mathrm{RPF}}_{\max}-{\mathrm{RPF}}_{\min}}{\mathrm{RPF}-{\mathrm{RPF}}_{\min}}$$

where DF, MC, RQ, PNEC, HQ, and RPF represent the pollutant’s detection frequency, mean concentration, ecological risk quotient, predicted no-effect concentration, non-carcinogenic risk quotient, and relative potency factor; max and min subscripts denote the maximum and minimum values of the corresponding indicator; and W_1_ = W_2_ = W_3_ = W_4_ = 1, W_5_ = W_6_ = 2. Sensitivity analysis confirmed that the core finding, namely that BPAP emerged as the highest-priority contaminant, remained robust when the health-to-other indicator weight ratio exceeded 1.3:1 (Figure S3).

#### 2.3.3. Source Apportionment Model: Enhanced Positive Matrix Factorisation with Dual-Media Coupling (PMF-DMC)

For spatially resolved source attribution, an enhanced Positive Matrix Factorisation model with Dual-Media Coupling (PMF-DMC) was applied using Python software (version 3.14, Python Software Foundation, Wilmington, DE, USA) [46]. This model integrates experimentally determined sediment–water partition coefficients (K_d_) to apply phase-specific weight corrections, enabling simultaneous source apportionment across water and sediment.

Prior to modelling, data were prepared from 20 valid water–sediment paired samples. Concentrations were standardised to ng/m^3^ for cross-media comparability. The optimal number of source factors (*p* = 4) was determined by minimising the deviation between observed and theoretical Q-values. A dual-media weighting factor, calculated as the mean aqueous-phase fractional contribution for key BPs, yielded final phase weights of 0.73 (water) and 0.27 (sediment) (The details in Text S3). To assess the robustness and stability of the four-factor solution given the sample size (n = 20), a comprehensive uncertainty analysis was performed using bootstrap resampling (100 iterations) in “Robust” mode to minimise sensitivity to outliers (Figure S4a). The model solution demonstrated high stability, as evidenced by several key diagnostics: (1) the base-run source contributions fell within the 95% confidence intervals of the bootstrap distributions, with relative standard deviations (RSD) ranging from 5.1% to 13.2%; (2) factor profile reproducibility was high, with mapping rates of 94–99% for the four factors across 97 accepted runs (Figure S4b,c); and (3) a displacement (DISP) analysis showed zero factor swaps (dQmax = 4%), confirming a unique and rotationally well-defined solution. Uncertainty diagnostics are presented in Figure S4 of the Supporting Information.

## 3. Results

### 3.1. Inadequacy of the Steady-State Paradigm: Observed Contradictions

**Figure 2:**
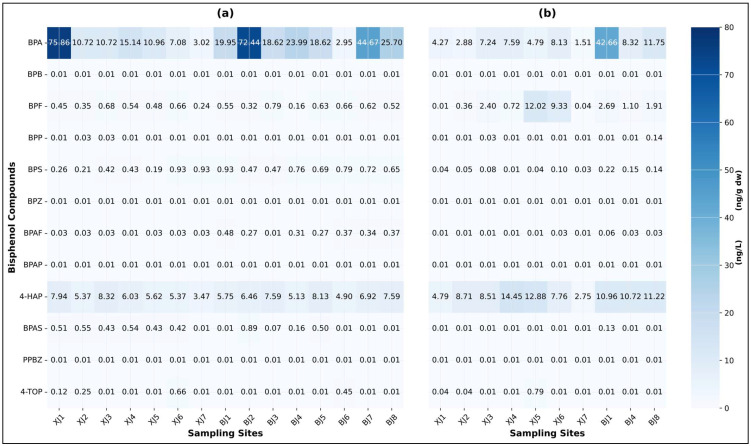
Bisphenol compounds log10-transformed concentrations in water and sediment from the Xijiang and Beijiang River Basins. (**a**) Measured concentrations in water bodies. (**b**) Measured concentrations in sediments.

**Figure 3:**
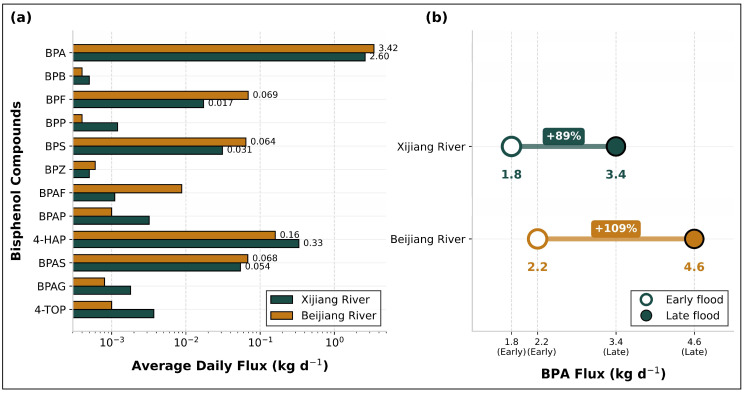
Comparison of pollution load fluxes of Bisphenol compounds between the Xijiang and Beijiang rivers. (**a**) Average daily flux of various bisphenol compounds. (**b**) BPA flux during early and late flood seasons.

**Figure 4:**
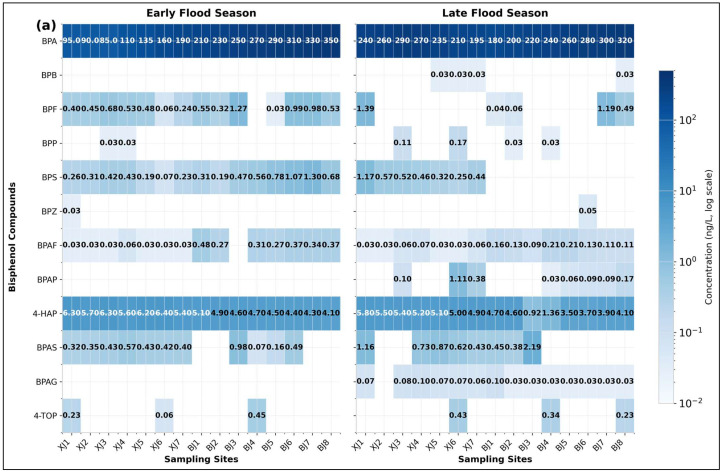

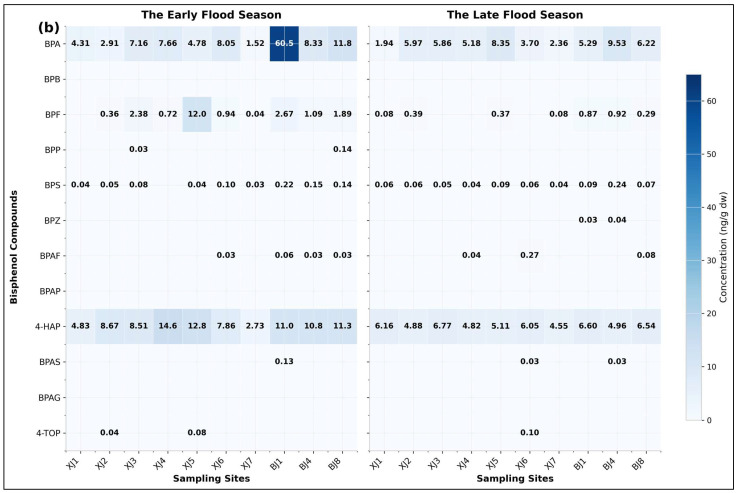
Monitoring of bisphenol compounds in water and sediments of the Xijiang and Beijiang Rivers across flood seasons. (**a**) In water. (**b**) In sediments.

A baseline assessment using conventional steady-state metrics revealed fundamental contradictions that challenge static risk assessment approaches. Spatiotemporal non-stationarity. BPs were detected in all water and sediment samples, confirming pervasive contamination. BPA was the dominant congener, accounting for 88.01% of total aqueous ΣBPs and 49.08% of sediment-associated ΣBPs. Pollution burden exhibited significant spatial stratification linked to land use: the industrialised Beijiang River carried a mean aqueous BPA concentration 1.81 times higher and a total bisphenol flux 22% greater than the agriculturally dominated Xijiang River (Figure 2 and Figure 3a). A pronounced temporal shift was also observed between the early and late flood seasons, with aqueous ΣBPs increasing 3.8-fold while sedimentary ΣBPs declined by 50.4% (*p* < 0.01) (Figure 4 and Figure S5). Concurrently, BPA flux in the Beijiang River increased by 133%, a rise far exceeding the proportional change in discharge. This decoupling of aqueous enrichment from sediment depletion constitutes field-validated evidence for flood-induced sediment remobilisation acting as a dynamic secondary source (Figure 3b).

**Figure 5:**
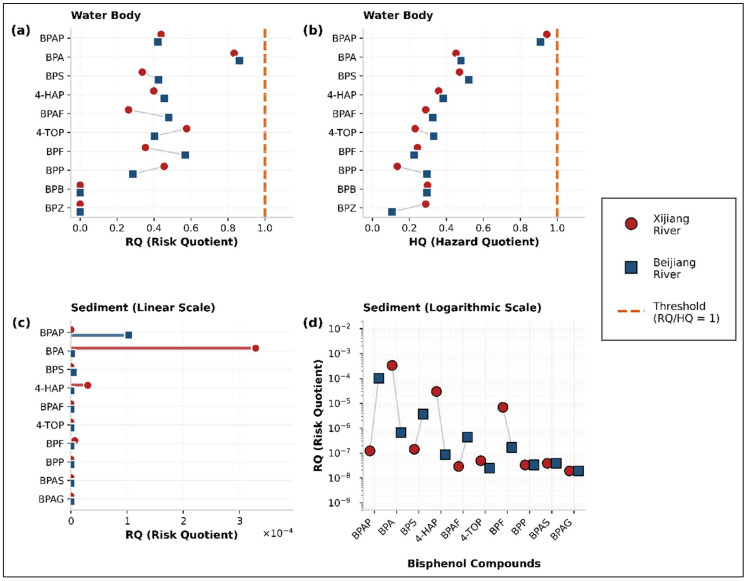
Risk quotient (RQ) and hazard quotient (HQ) of bisphenol compounds in the Xijiang and Beijiang River Basins. (**a**) Water body RQ values. (**b**) Water body HQ values. (**c**) Sediment RQ values (linear scale). (**d**) Sediment RQ values (logarithmic scale).

Discordance in conventional risk metrics. These dynamics exposed the limitations of single-metric, concentration-centric risk assessment. Conventional ecological RQ analysis identified BPA as the only compound of medium concern. In contrast, HQ assessment, which is pertinent to the watershed’s drinking water source function, flagged BPAP as the highest-priority compound (Figure 5). This paradox, whereby BPA dominates in environmental abundance, but BPAP prevails in toxicological potency, highlights the “regrettable substitution” dilemma and underscores the inadequacy of static prioritisation frameworks.

The collinearity challenge. Furthermore, initial attempts to quantitatively diagnose the underlying sediment-release process were confounded by severe collinearity between key environmental drivers, DOC and water T (r = 0.98, *p* < 0.001). This prevented the statistical separation of their individual and synergistic effects, creating a mechanistic “black box”. Collectively, these observed contradictions, including spatiotemporal non-stationarity, discordance between conventional risk metrics, and unresolved process collinearity, point to the inadequacy of the steady-state paradigm and call for a new modelling approach grounded in process-based understanding.

### 3.2. The Environmental Condition Index (ECI): Quantifying Synergistic Process Control

**Figure 6:**
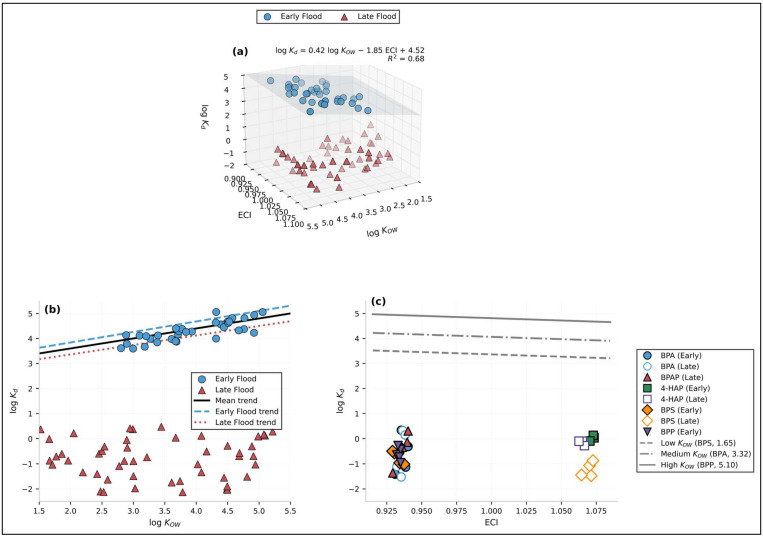
Relationship of logK_d_ with ECI and log K_OW_ for target compounds during early and late flood seasons. (**a**) Three-dimensional scatter plot showing the dependence of logK_d_ (distribution coefficient, L/kg) on both ECI (electrochemical index) and logK_OW_ (octanol–water partition coefficient). The fitted regression plane (translucent blue surface) represents the multivariate linear relationship: logK_d_ = 0.42 logK_OW_ − 1.85 ECI + 4.52 (R^2^ = 0.68, n= 85). Blue circles denote the early flood season; red triangles denote the late flood season. (**b**) Two-dimensional projection of logK_d_ versus logK_OW_. The solid black line represents the mean trend across all samples; the blue dashed line and red dotted line represent the early flood and late flood trends, respectively. The vertical separation between early and late flood clusters indicates that flood seasonality significantly influences the distribution behaviour independent of hydrophobicity. (**c**) Two-dimensional projection of logK_d_ versus ECI, with individual compounds grouped by their logK_OW_ ranges. Filled symbols = early flood; open symbols = late flood. The three grey reference lines represent the theoretical logK_d_ values predicted at low (BPS, logK_OW_ = 1.65, dashed), medium (BPA, logK_OW_ = 3.32, dash-dotted), and high (BPP, logK_OW_ = 5.10, solid) hydrophobicity levels, illustrating how increasing logK_OW_ elevates the baseline logK_d_ while ECI exerts a negative effect.

The developed ECI successfully resolved the DOC–T collinearity by encoding their synergistic influence into a single, process-based metric. The subsequent partitioning model (Figure 6) yielded a robust prediction of logK_d_ dynamics (R^2^ = 0.68). The model’s parameters, derived from theoretically constrained regression and validated via bootstrap resampling, are central to its interpretation. The positive coefficient for logK_ow_ (b = +0.42; 95% CI: 0.32, 0.52) confirms the role of compound hydrophobicity in sediment adsorption under baseline conditions. Crucially, the strong negative coefficient for the ECI (c = −1.85; 95% CI: −2.26, −1.44) quantitatively demonstrates that transient environmental forcing, not intrinsic hydrophobicity, governs phase partitioning during hydrologically active periods. This coefficient mechanistically captures the synergistic desorption effect: concurrent elevated T (primary thermodynamic driver) and high DOC (secondary complexing agent) significantly reduce sediment–water affinity, promoting contaminant release. The ECI thus functions as a validated “process proxy,” transforming the qualitative understanding of sediment as a “sink” into a quantitative, condition-responsive model for forecasting its activation as a dynamic “source” [47].

### 3.3. The Dual-Media ToxPi Model: Integrated Risk Prioritisation Reversals

**Figure 7:**
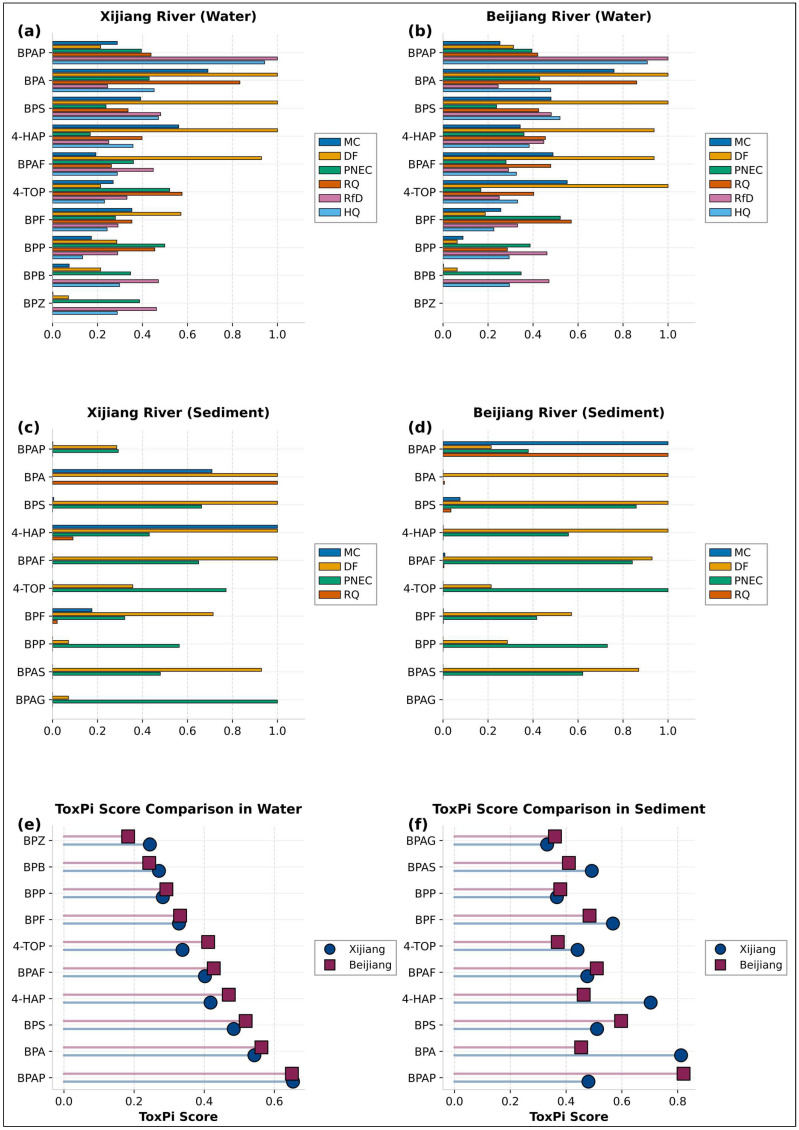
The normalised values of each indicator and ToxPi scores for bisphenol compounds in the water–sediment system of the Xijiang and Beijiang River Basins. (**a**) Standardised scores for five indicators in Xijiang River water. (**b**) Standardised scores for five indicators in Beijiang River water. (**c**) Standardised scores for four indicators in Xijiang River sediments. (**d**) Standardised scores for four indicators in Beijiang River sediments. (**e**) Comparison of ToxPi scores in water between the two basins. (**f**) Comparison of ToxPi scores in sediments between the two basins.

The extended ToxPi model, integrating six standardised risk dimensions across water and sediment media, produced a holistic risk ranking that fundamentally challenged concentration-centric assessments (Tables S9–S12; Figure 7). When multi-dimensional attributes were synthesised, specifically detection frequency, mean concentration, inherent toxicity (PNEC, RPF), and realised risk (RQ, HQ), BPAP emerged as the highest-priority contaminant, followed by BPA.

This priority reversal is a direct output of the model’s integrative design. The double weighting of health-related indicators explicitly prioritises compounds with high intrinsic health hazards, reflecting the watershed’s drinking water source function. Sensitivity analysis confirmed the robustness of this finding; the BPAP > BPA reversal remained stable within a plausible decision-space. This demonstrates that the model’s output provides a transparent, multi-criteria basis for management prioritisation that moves beyond ambient concentration alone. The shift emerges from coupling the process diagnostics of the ECI with the integrative assessment of ToxPi. The ECI quantifies that high-ECI conditions increase the potential for release of sediment-bound contaminants. Within this context of amplified exposure, the ToxPi model identifies that high-potency compounds like BPAP experience a greater relative increase in their integrated risk score. Thus, under dynamic conditions that activate sediments as a “risk amplifier,” the relative importance of high-toxicity compounds is elevated compared to static, concentration-driven paradigms.

This mechanistic link can be conceptualised as a two-stage amplification process: first, the ECI model quantifies a condition-dependent exposure multiplier. For instance, the model coefficient (c = −1.85) indicates that a unit increase in ECI leads to a nearly two-order-of-magnitude decrease in logK_d_, substantially increasing the aqueous-phase concentration of sediment-associated contaminants. Second, the ToxPi model acts as a potency-dependent risk integrator. For a compound like BPAP, which has a high inherent health toxicity (low RfD, high RPF), even a modest increase in aqueous concentration (driven by high ECI) results in a disproportionately large increase in its HQ and, consequently, its overall ToxPi score. In contrast, for a high-concentration compound like BPA, the same absolute increase in concentration has a smaller relative impact on its already high exposure-driven scores. Thus, dynamic environmental conditions (high ECI) differentially amplify the integrated risk of high-potency contaminants, leading to the observed priority reversal.

The robustness of our findings provides further validation of the significance and physical meaning underlying the ECI’s weighting scheme. The strong negative correlation between the ECI and log K_d_ (c = −1.85, *p* < 0.05, Figure 6a) represents a physically meaningful result, demonstrating that the model’s weight assignment successfully captures a real environmental process: elevated temperature and DOC concentrations promote the desorption of bisphenol compounds into the water phase. Moreover, the subsequent ToxPi model revealed that the priority ranking of BPAP as the highest-risk compound remained stable across different weighting schemes (Figure S3). This stability indicates that the overall conclusions of the study are not sensitive to minor variations in specific weight choices within a reasonable range, thereby reinforcing the reliability of the ECI as a process-informed diagnostic tool.

### 3.4. An Integrated Process-Informed Dynamic Risk Framework

**Figure 8:**
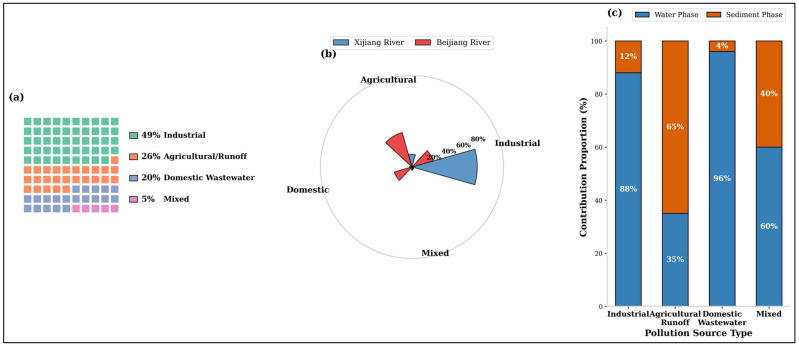
Source factor profiles and watershed-scale contributions of bisphenol compounds resolved by the PMF-DMC model). (**a**) Source contribution profile for the Foshan section of the Pearl River Basin (each square represents 1%). (**b**) Comparison of pollution source contribution proportions between the Xijiang and Beijiang River basins. (**c**) Contribution characteristics of different pollution source types to the water and sediment phases.

**Figure 9:**
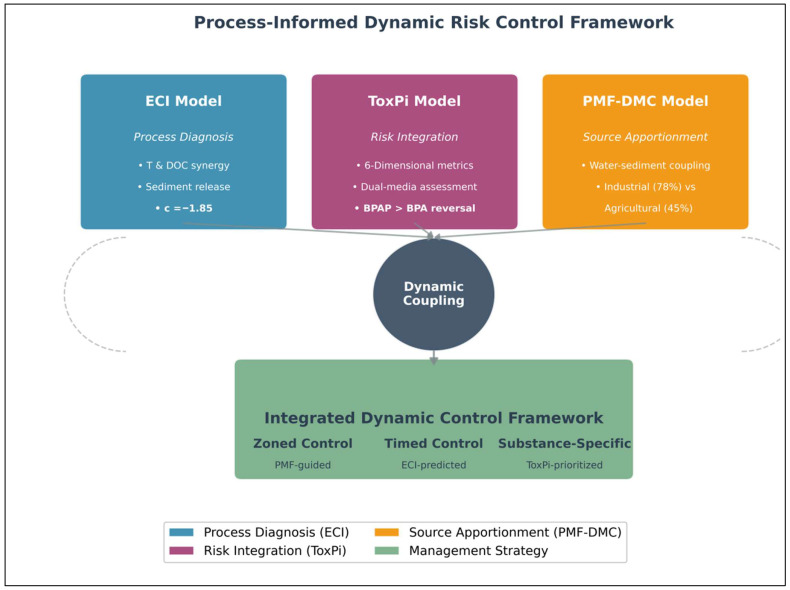
Integrated dynamic risk control framework conceptual diagram.

The synergistic integration of the ECI and ToxPi models (Figure 8) with spatially resolved source apportionment (via PMF-DMC) yields a unified framework that transcends steady-state assumptions. This framework operates on two synergistic pillars (Figure 9):

Process Diagnosis and Forecasting (ECI Model): Quantifies the environmental propensity (high ECI) for activating sedimentary “risk reservoirs,” predicting when (e.g., flood season) and why (synergistic T and DOC effects) exposure spikes occur.

Holistic Risk Integration and Prioritisation (Dual-Media ToxPi Model): Synthesises multi-dimensional attributes to identify which contaminants (e.g., BPAP) pose the greatest integrated threat, irrespective of ambient concentration, thereby flagging high-hazard substances within the dynamic reservoirs diagnosed by ECI.

Their coupling completes the causal “process–risk” chain, transforming risk assessment from a static inventory into a predictive early-warning system. Building on the “source-process-risk” coupling framework, we propose a targeted, three-dimensional management strategy:

1. Spatial Zoning (Guided by PMF-DMC): Implement differentiated control measures based on dominant source patterns. For the industrial-dominated Beijiang River, enforcement should focus on industrial wastewater treatment upgrades and pollution discharge permits for key sectors (e.g., plastics, electronics). For the agriculture-influenced Xijiang River, management should shift towards non-point source control and regulating agricultural runoff.
2. Temporal Phasing (Informed by ECI Forecasting): Adapt monitoring and early-warning schedules to hydrological dynamics. Prior to and during the monsoon flood season (high ECI period), environmental agencies should intensify surveillance of sediment and water quality, particularly for high-priority, sediment-associated contaminants like BPAP. This allows for pre-emptive measures to mitigate peak exposure events.
3. Substance-Specific Prioritisation (Driven by ToxPi): Update local regulatory lists and standards based on dynamic risk rather than static concentration. Given its top-ranked ToxPi score, BPAP is recommended as a high-priority candidate for further monitoring and risk assessment in the region. The development of a health-based water quality benchmark or guideline value for BPAP could be considered as a longer-term policy goal, contingent upon the availability of additional toxicological and exposure data, as well as formal regulatory review. Such a step would help to move beyond the current sole focus on BPA, but should be supported by a more comprehensive evidence base.

## 4. Discussion

### 4.1. Advancing Dynamic Risk Assessment Through an Integrated Framework

This study advances ecological modelling by formally integrating three distinct model classes, a process diagnostic model (ECI), a multi-criteria risk integration model (ToxPi), and a spatially resolved source apportionment model (PMF-DMC), into a unified framework. This synthesis directly addresses a critical shortcoming in conventional approaches, which often assess environmental processes, risk, and source attribution in isolation [5,48]. Within our framework, pervasive driver collinearity is uniquely resolved through a mechanistic composite index; multi-criteria risk integration is extended to dual-media systems; and source attribution is employed to contextualise outputs for targeted management. By shifting the paradigm from analysing isolated components to simulating their interactive dynamics and emergent risk consequences, this integrated approach offers a more holistic understanding of contaminant behaviour in complex watersheds.

To contextualise the measured concentrations and risk patterns observed in this study, we compared our findings with those reported for other major river systems globally. The mean total bisphenol concentration in the Foshan reach of the Pearl River Basin (163 ng/L in water; 17.5 ng/g dw in sediment) was substantially lower than in urban-impacted systems such as the Guangzhou urban waterways (4720 ng/L in water; 117 ng/g dw in sediment; [38]) and the Taihu Lake Basin (324 ng/L in water; 31.9 ng/g dw in sediment; [49,50]). However, it was comparable to, or slightly higher than, less urbanised systems like the Lanzhou section of the Yellow River (74.6 ng/L in water; 35.2 ng/g dw in sediment) and the Liao River Basin (63 ng/L in water; 0.34 ng/g dw in sediment; [51]). Regarding individual congeners, the concentration of BPA, the dominant bisphenol in this study (mean 71.8 ng/L in water), was comparable to levels reported for the Yangtze River Estuary (mean 89.2 ng/L; [52]) and the Han River in South Korea (mean 54.3 ng/L; [53]), yet considerably lower than those in the Pearl River Delta urban waterways (mean 890 ng/L; [54]). Notably, our finding that BPA dominated ecological risk (highest RQ) while BPAP dominated human health risk (highest HQ) is consistent with the emerging recognition that alternative bisphenols, such as BPAP and BPS, can pose higher health risks than BPA. This is primarily due to their much lower reference doses, despite their lower environmental concentrations [55,56,57]. This pattern has also been observed in the Yangtze River Estuary [52] and in Korean river systems [53], suggesting that the shift in priority from BPA to BPAP may be a broader phenomenon in dynamic river systems receiving both industrial and domestic inputs. In summary, these comparisons underscore that while absolute concentrations vary widely across systems, the relative risk ranking and the emergence of alternative bisphenols as priority compounds follow consistent patterns, warranting coordinated monitoring and risk management efforts.

### 4.2. Limitations and Future Perspectives

Despite the valuable insights provided by this study on the occurrence, source apportionment, and risk assessment of BPs in the Pearl River system, several limitations must be acknowledged to guide future investigations. First, the generalisability of the current findings is constrained by the sampling strategy. The study is limited to a single wet season and a specific geographic region; thus, future research should incorporate year-round sampling across multiple hydrological years to capture seasonal and interannual variability. Additionally, expanding the study scope to include other river basins and a broader spectrum of emerging contaminants, beyond BPs, would further validate the transferability of the proposed ECI-PMF framework [58,59].

Second, the study’s reliance on targeted chemical analysis introduces inherent constraints. While the non-targeted screening (NTA) approach was employed for initial characterisation, the quantitative risk assessment was performed exclusively on a predefined list of 12 BPs. This focus may overlook the synergistic effects of unidentified co-occurring contaminants and their metabolites [60]. Future work should integrate high-resolution mass spectrometry (HRMS)-based suspect and non-targeted screening with effect-directed analysis (EDA) to provide a more holistic evaluation of the total toxic burden. Furthermore, the static risk assessment performed here does not account for the dynamic bioaccumulation and transformation kinetics of BPs in aquatic organisms under fluctuating environmental conditions.

Finally, methodological refinements are warranted to enhance the robustness of the source apportionment and risk characterisation. The PMF model’s performance is sensitive to input data quality and the number of factors specified; future studies should incorporate Monte Carlo-based uncertainty propagation and multi-model intercomparison (e.g., PCA-MLR, UNMIX) to strengthen source attribution confidence. Similarly, the ecological risk assessment relied on species sensitivity distributions (SSDs) derived from acute toxicity data, which may underestimate chronic, sublethal effects. Future research should prioritise chronic SSD derivation and in situ bioassay validation to improve ecological relevance [61]. Collectively, addressing these limitations will not only refine our understanding of BP contamination dynamics but also establish a more transferable and scientifically robust framework for prioritising emerging contaminants in complex aquatic environments.

### 4.3. Revealing Altered Risk Priorities and Challenging Conventional Paradigms

A pivotal finding of this study is the identification of a critical risk priority reversal in the Pearl River Basin. Contrary to conventional frameworks that predominantly identify BPA as the primary risk driver due to its high environmental abundance [37,38,49], our integrated framework reveals BPAP as the highest-priority control pollutant. This discrepancy stems from the traditional reliance on concentration-centric metrics, such as the ecological RQ based solely on PNEC and MEC. Such conventional approaches, as highlighted by Ye et al. (2023, 2024) [10,11], systematically underestimate the risks posed by low-abundance but high-toxicity substitutes, a phenomenon often termed “regrettable substitution” [4,62,63]. Our novel ECI effectively resolves the multicollinearity between key environmental drivers (e.g., DOC and T) that often confounds traditional partitioning models [9,25,26], providing a robust diagnostic tool for dynamic systems. Subsequently, our extended dual-media ToxPi model directly challenges the static paradigm by assigning higher weight to health hazard attributes [13,14], thereby offering a more comprehensive and ecologically relevant assessment that accounts for the intrinsic toxicological potency of emerging contaminants like BPAP. This demonstrates the framework’s capacity to uncover latent threats overlooked by conventional assessments.

### 4.4. Framework Transferability, Applicability, and Adaptive Governance

Designed for broad transferability, the framework’s adaptation requires contextual calibration: the ECI can be reconfigured with local environmental variables, and the ToxPi’s dimensions and weights can be adjusted to match specific management objectives. While its application in the Pearl River Basin successfully identified a crucial “risk priority reversal,” transferability to other watersheds is contingent on the availability of high-resolution temporal data. Unlike static models, which often require only snapshot sampling or limited historical data [5,48,64], our process-informed approach demands continuous or high-frequency monitoring of hydrological events and key environmental parameters. This requirement for robust temporal datasets, which may not be readily available in data-scarce regions [19,48], could potentially limit its immediate application. Future research should explore methods for integrating sparse data or developing proxy indicators to enhance the framework’s applicability in such contexts. Nevertheless, the framework’s outputs provide a direct scientific foundation for adaptive governance. The proposed “Zoned–Timed–Substance-Specific” management strategy exemplifies how integrated modelling results can be translated into actionable management plans, reframing the central management question from “What is the current risk?” to “Under what future conditions will latent risks materialise?” Operationalising this strategy requires embedding the framework’s predictive outputs into decision-support systems, enabling anticipatory responses and strengthening watershed resilience to dynamic environmental change [27,48,65].

## 5. Conclusions

This study developed and validated an integrated, process-informed modelling framework to advance risks assessment beyond static, concentration-centric paradigms for dynamic watersheds. The framework formally couples a novel ECI to resolve driver collinearity and quantify transient environmental forcing, with an extended dual-media ToxPi for holistic, multi-criteria risk integration across compartments.

The key finding was a risk priority reversal identified in the Pearl River Basin case study. When process-driven exposure amplification (via ECI) was integrated with multi-attribute hazard assessment (via ToxPi), BPAP emerged as the highest-priority control pollutant, contrary to steady-state conclusions based solely on the relative abundance of BPA. This demonstrates the framework’s critical capacity to uncover latent threats that conventional assessments systematically overlook, directly addressing the “regrettable substitution” dilemma.

The framework provides a transferable methodological advance. Its core innovation, using a mechanistic composite index to isolate synergistic process control, can be reconfigured with locally relevant environmental drivers. Similarly, the dual-media ToxPi can be adapted to specific management objectives by adjusting its risk dimensions and weights. Synthesised into a “Zoned–Timed–Substance-Specific” strategy, this work shifts the central management question from “What is the risk now?” to “Under what future conditions will latent risks materialise?”. Future studies should investigate the removal efficiency of conventional and advanced drinking water treatment processes for the priority bisphenol compounds identified in this study, particularly BPAP, in order to refine health risk estimates for treated drinking water and provide more realistic guidance for water safety management.

## Data Availability

The raw data supporting the conclusions of this article will be made available by the authors on request.

## Supplementary Materials

The following supporting information can be downloaded at https://www.mdpi.com/article/10.3390/toxics14050448/s1, Figure S1: Determination of optimal weighting coefficients for dissolved organic carbon (DOC) and temperature in the Environmental Co-occurrence Index (ECI) model through multi-criteria sensitivity analysis. Figure S2: Bootstrap Distributions and Residual Diagnostics of the ECI Partitioning Model (with 10,000 iterations). Figure S3: ToxPi Model Sensitivity Analysis: Weighting Scheme Comparison. Figure S4: Bootstrap uncertainty assessment of the PMF-DMC model. (a) Distributions of source contribution percentages across 100 bootstrap runs (boxplots with 95% confidence intervals), with base-run values indicated by stars (★). Three runs were rejected due to Q-value decrease >10%, yielding 97 accepted runs. Relative standard deviations (RSD) range from 5.1% to 13.2%. (b) Factor profile stability: base-run profiles (solid lines with squares) overlaid with bootstrap means (bars) and 95% confidence intervals (error bars) for each of the four resolved sources across 10 bisphenol compounds. (c) Factor mapping reproducibility matrix (mapping threshold: Pearson r = 0.6) showing mapping rates of 94–99% among 97 accepted bootstrap runs. DISP analysis: zero factor swaps, dQmax = 4%. The minor off-diagonal elements (1–2%) for the Mixed factor reflect its inherent compositional overlap with industrial and agricultural sources. Figure S5: Comparison of average bisphenol compound concentrations in sediment and water bodies. Table S1: Longitude, latitude, name, and type of the sample sites. Table S2: Sampling Section Information for Watersheds. Table S3: Physical and Chemical Parameters of Watershed Sampling Sections. Table S4: Physiochemical properties of the Bisphenol compounds in this study. Table S5: Sediment–Water Partition Coefficients (K_d_) of BPs in Early and Late Flood Season. Table S6: Hydrological Parameters and Flow Calculations at Monitoring Cross-Sections. Table S7: Calculated DOC Concentrations for Each District/County. Table S8: The RfD values and PNEC values for ecological risk assessment of BPs. Table S9: The normalised values of each indicator and ToxPi scores for bisphenol compounds in the water bodies of the Xijiang River Basin. Table S10: The normalised values of each indicator and ToxPi scores for bisphenol compounds in the water bodies of the Beijiang River Basin. Table S11: The normalised values of each indicator and ToxPi scores for bisphenol compounds in the sediments of the Xijiang River Basin. Table S12: The normalised values of each indicator and ToxPi scores for bisphenol compounds in the sediments of the Beijiang River Basin. Text S1: Comprehensive Water and Sediment Sampling Protocol. Text S2: Chemical Analysis and Quality Assurance/Quality Control (QA/QC). Text S3: Calibration of Dual-Media Weight (w) for the PMF-DMC Model.
