# Supplementary material for: Beyond Steady-State: An Integrated Framework Unveils BPAP as the Highest-Risk Bisphenol in a Dynamic River System
Source: Toxics. 2026 May 20;14(5):448. doi: 10.3390/toxics14050448 (PMC13211460; doi:10.3390/toxics14050448)
Supplement: Supplementary file 1 [file toxics-14-00448-s001.zip › toxics-4274806-supplementary.pdf]

Supplementary Materials

MANUSCRIPT TITLE: Beyond Steady-State: An Integrated Framework Unveils BPAP as the Highest-Risk Bisphenol in a Dynamic River System

AUTHORS: Zheng Zhang, Lulu Zhang, Jingru Zhang, Lingyun Yu, Yujun Tong, Qiusen Huang, Yueping Zhu, Wenyu Xie, Dongpo Liu

ADDRESS: Laboratory of Risk Assessment and Control of New Pollutants, Guangdong Provincial Academy of Environmental Sciences, Guangzhou 510045, China

School of Environmental Science and Engineering, Guangdong University of Petrochemical Technology, Maoming 525000, China

TABLES: 12

FIGURES: 5

TEXT: 3

NO. OF PAGES: 23

Table S1 Longitude, latitude, name, and type of the sample sites.

Site	Longitude	Latitude	Name	Type
1	112.81	23.15	XJ1	Provincial Control, Drinking Water Source Protection, Xi jiang Entry Point in Foshan
2	112.84	23.01	XJ2	Provincial Control
3	112.89	22.91	XJ3	Drinking Water Source Protection
4	112.90	22.89	XJ4	National Control, Cangjiang Tributary Outlet
5	113.01	22.81	XJ5	Drinking Water Source Protection
6	113.06	22.81	XJ6	Provincial Control, Drinking Water Source Protection
7	113.22	22.72	XJ7	Provincial Control, Drinking Water Source Protection, Xi jiang Exit Point in Foshan
8	113.90	23.49	BJ1	National Control, Provincial Control, Beijiang Entry Point in Foshan
9	112.90	23.37	BJ2	Provincial Control, Drinking Water Source Protection
10	112.82	23.15	BJ3	National Control, Provincial Control
11	112.94	23.12	BJ4	Drinking Water Source Protection
12	113.03	23.02	BJ5	Provincial Control, Drinking Water Source Protection
13	113.05	22.92	BJ6	Provincial Control, Drinking Water Source Protection
14	113.19	22.89	BJ7	Drinking Water Source Protection
15	113.26	22.90	BJ8	National Control, Beijiang Exit Point in Foshan

Table S2 Sampling section information for watersheds.

Sampling Section	Early Flood Season			Late Flood Season		
	Median Flow Velocity (m/s)	Depth（m）	Width（m）	Median Flow Velocity (m/s)	Depth（m）	Width（m）
XJ1	0.216	11	300	0.153	15	300
XJ2	0.148	10	300	0.163	12	300
XJ3	0.062	12	400	0.180	15	400
XJ4	0.066	15	500	0.163	15	500

XJ5	0.021	10	400	0.064	12	400
XJ6	0.109	12	300	0.076	15	300
XJ7	0.117	15	300	0.068	16	300
BJ1	0.073	13	500	0.117	15	500
BJ2	0.183	15	300	0.117	15	300
BJ3	0.111	12	300	0.133	14	300
BJ4	0.102	15	140	0.073	13	140
BJ5	0.426	8	60	0.058	10	60
BJ6	0.055	14	400	0.053	15	400
BJ7	0.168	15	400	0.196	18	400
BJ8	0.090	11	50	0.054	17	50

Table S3 Physical and chemical parameters of watershed sampling sections.

Sampling Section	Early Flood Season								Late Flood Season							
	Dissolved Oxygen (mg/L)	pH	Conductivity (μ s/cm)	Ammonia Nitrogen (mg/L)	Temperature (° C)	Salinity (‰)	daily precipitation amount（mm）	Dissolved Organic carbon Concentration（mg/L）	Dissolved Oxygen (mg/L)	pH	Conductivity (μ s/cm)	Ammonia Nitrogen (mg/L)	Temperature (° C)	Salinity (‰)	daily precipitation amount（mm）	Dissolved Organic carbon Concentration（mg/L）
XJ1	8.38	6.87	247	<0.2	25.2	0.08	3.96	3.3	6.85	7.83	216	<0.2	29.2	0.07	21.51	3.3
XJ2	8.66	6.88	241	<0.2	25.5	0.07	3.96	1.8	7.19	8.01	277	<0.2	29.1	0.06	21.51	1.8
XJ3	8.56	6.91	267	<0.2	25.3	0.08	3.96	1.8	7.46	7.99	273	<0.2	29.8	0.08	21.51	1.8
XJ4	8.3	6.95	258	<0.2	25.4	0.07	3.96	1.8	7.55	8.05	276	<0.2	29.9	0.06	21.51	1.8
XJ5	8.48	7.01	261	<0.2	25.5	0.06	3.96	2.8	6.35	8.30	283	<0.2	32.3	0.07	21.51	2.8
XJ6	8.86	7.01	250	<0.2	25.5	0.07	3.96	2.5	7.45	8.40	290	<0.2	30.3	0.07	21.51	2.5
XJ7	8.6	7.00	263	<0.2	25.5	0.07	3.96	2.5	6.74	8.48	284	<0.2	29.7	0.06	21.51	2.5
BJ1	6.47	7.83	163	<0.2	30.2	0.06	1.46	3.3	7.11	7.88	210	<0.2	32.1	0.06	22.57	3.3
BJ2	7.04	7.92	173	<0.2	31.4	0.04	1.46	3.3	6.77	7.81	204	<0.2	31.7	0.07	22.57	3.3
BJ3	8.23	6.92	195	<0.2	25.3	0.05	1.46	3.3	6.66	7.85	208	<0.2	29.1	0.06	22.57	3.3
BJ4	6.01	7.84	221	<0.2	27.7	0.07	1.46	2.8	6.86	7.81	187	<0.2	27.6	0.06	22.57	2.8
BJ5	5.94	7.72	221	<0.2	27.7	0.07	1.46	3	6.78	7.85	193	<0.2	28.5	0.06	22.57	3
BJ6	6.00	7.93	234	<0.2	28.3	0.07	1.46	2.5	6.22	7.75	188	<0.2	30.2	0.06	22.57	2.5
BJ7	6.13	7.84	235	<0.2	28.1	0.06	1.46	2.5	6.19	7.68	196	<0.2	29.7	0.07	22.57	2.5
BJ8	6.21	7.92	221	<0.2	27.2	0.07	1.46	2.5	6.29	7.79	218	<0.2	28.6	0.06	22.57	2.5

Table S4 Physiochemical properties of the Bisphenol compounds in this study.

Bisphenol compounds	Abbreviation	CAS number	Chemical formula
Bisphenol A	BPA	80-05-7	$C_{15}H_{16}O_2$
Bisphenol B	BPB	77-40-7	$C_{16}H_{18}O_2$
Bisphenol F	BPF	620-92-8	$C_{13}H_{12}O_2$
Bisphenol P	BPP	2167-51-3	$C_{21}H_{20}O_2$
Bisphenol S	BPS	80-09-1	$C_{12}H_{10}O_4S$
Bisphenol Z	BPZ	843-55-0	$C_{18}H_{22}O_2$
Bisphenol AF	BPAF	1478-61-1	$C_{15}H_{10}F_6O_2$
Bisphenol AP	BPAP	1571-75-1	$C_{19}H_{16}O_2$
4'-Hydroxyacetophenone	4-HAP	99-93-4	$C_8H_8O_2$
Bisphenol AS	BPAS	108494-70-0	$C_{19}H_{15}NO_3$
Bisphenol AG	BPAG	1844-01-5	$C_{17}H_{18}O_8$
4-tert-Octylphenol	4-TOP	140-66-9	$C_{14}H_{22}O$

TableS5 Sediment–water partition coefficients (K_d) of BPs in early and late flood season.

Bisphenol compounds	Early flood Season (Water, ng/L)	Early flood Season (Sediment, ng/g dw)	K_d (L/kg)	Late flood Season (Water, ng/L)	Late flood Season (Sediment, ng/g dw)	K_d (L/kg)
BPA	25.9	11.70	0.452	118	5.44	0.0461
BPB	<0.01	<0.01	-	0.01	<0.01	-
BPF	0.69	2.21	3.203	1.56	0.30	0.192
BPP	<0.01	0.02	-	0.02	<0.01	-

BPS	0.56	0.08	0.143	1.70	0.08	0.0471
BPZ	<0.01	<0.01	-	0.01	<0.01	-
BPAF	0.17	0.02	0.118	0.09	0.02	0.222
BPAP	<0.01	<0.01	-	0.18	0.04	0.222
4-HAP	6.29	9.31	1.480	5.26	5.64	1.072
BPAS	0.33	0.02	0.0606	2.49	0.02	0.00803
BPAG	<0.01	<0.01	-	0.05	<0.01	-
4-TOP	0.09	0.01	0.111	0.07	0.01	0.143

TableS6 Hydrological parameters and flow calculations at monitoring cross-sections.

Sampling Section	Median Flow Velocity (m/s)	Depth (m)	Width (m)	Cross-sectional Flow Rate *Q* (m^3 /s)
XJ1	0.1845	13	300	719.55
XJ2	0.1555	11	300	513.15
XJ3	0.121	13.5	400	653.4
XJ4	0.1145	15	500	858.75
XJ5	0.0425	11	400	187
XJ6	0.0925	13.5	300	374.625
XJ7	0.0925	15.5	300	430.125
BJ1	0.095	14	500	665
BJ2	0.15	15	300	675
BJ3	0.122	13	300	475.8
BJ4	0.0875	14	140	171.5
BJ5	0.242	9	60	130.68
BJ6	0.054	14.5	400	313.2
BJ7	0.182	16.5	400	1201.2
BJ8	0.072	14	50	50.4

TableS7 Calculated *DOC* concentrations for each district/county.

District	Dominant Land-Use Type	Avg. COD_{Mn} (mg/L)	Estimated *DOC* Range (mg/L)	Typical *DOC* Value (mg/L)	Key Influencing Factors
Chancheng	Central Urban Area	3.5	2.2 ~3.8	3.0	Domestic sewage, surface runoff
Nanhai	Industrial Zone	3.2	2.0 ~3.5	2.8	Industrial refractory organics
Shunde	Manufacturing Zone	2.9	1.8 ~3.2	2.5	Manufacturing discharge, water self-purification
Sanshui	Agricultural Area & River Confluence	3.7	2.5 ~4.2	3.3	Agricultural non-point source, flow mixing and accumulation
Gaoming	Ecological Conservation Area	2.4	1.2 ~2.5	1.8	Forest coverage, wetland purification

Table S8 The *RfD* values and *PNEC* values for ecological risk assessment of BPs.

Bisphenol compounds	*RfD*（ng/kg·d）	*PNEC*（μg/L）
BPA	500000	0.24

BPB	5000	1.35007
BPF	198000	5.44092
BPP	200000	0.05731
BPS	4000	12.88093
BPZ	6000	0.58774
BPAF	8000	1.01908
BPAP	0.1	0.49538
4-HAP	450000	55.09995
4-TOP	86400	0.03607

Table S9 The normalized values of each indicator and ToxPi scores for bisphenol compounds in the water bodies of the Xijiang River Basin.

Bisphenol compounds	MC	DF	PNEC	RQ	RfD	HQ	ToxPi score
BPAP	0.288	0.214	0.395	0.438	1.000	0.943	0.653
BPA	0.691	1.000	0.430	0.833	0.245	0.452	0.543
BPS	0.392	1.000	0.238	0.336	0.481	0.471	0.484
4-HAP	0.561	1.000	0.168	0.398	0.250	0.358	0.418
BPAF	0.193	0.929	0.360	0.262	0.448	0.288	0.402
4-TOP	0.270	0.214	0.521	0.576	0.331	0.231	0.338
BPF	0.353	0.571	0.280	0.353	0.291	0.243	0.328
BPP	0.173	0.286	0.499	0.455	0.290	0.134	0.282
BPB	0.074	0.214	0.347	0.000	0.471	0.298	0.271
BPZ	0.002	0.071	0.387	0.000	0.462	0.288	0.245

Table S10 The normalized values of each indicator and ToxPi scores for bisphenol compounds in the water bodies of the Beijiang River Basin.

Bisphenol compounds	MC	DF	PNEC	RQ	RfD	HQ	ToxPi score
BPAP	0.253	0.313	0.395	0.421	1.000	0.908	0.650
BPA	0.760	1.000	0.430	0.861	0.245	0.479	0.563
BPS	0.480	1.000	0.238	0.425	0.481	0.520	0.518
4-HAP	0.343	0.938	0.360	0.455	0.448	0.383	0.470
BPAF	0.489	0.938	0.280	0.479	0.291	0.326	0.427
4-TOP	0.552	1.000	0.168	0.403	0.250	0.331	0.411
BPF	0.257	0.188	0.521	0.570	0.331	0.226	0.331
BPP	0.089	0.063	0.387	0.286	0.462	0.294	0.292
BPB	0.002	0.063	0.347	0.000	0.471	0.295	0.243
BPZ	0.048	0.125	0.499	0.000	0.290	0.106	0.183

Table S11 The normalized values of each indicator and ToxPi scores for bisphenol compounds in the sediments of the Xijiang River Basin.

Bisphenol compounds	MC	DF	PNEC	RQ	ToxPi score
BPAP	0.02	0.286	163.993	1.22×10^{-7}	0.480
BPA	4.98	1.000	15.139	3.29×10^{-4}	0.812
BPS	0.05	1.000	352.517	1.42×10^{-7}	0.511
4-HAP	7.02	1.000	234.154	3.00×10^{-5}	0.703
BPAF	0.01	1.000	345.719	2.89×10^{-8}	0.476
4-TOP	0.02	0.357	408.035	4.90×10^{-8}	0.441
BPF	1.24	0.714	178.741	6.94×10^{-6}	0.568
BPP	0.01	0.071	302.030	3.31×10^{-8}	0.367
BPAS	0.01	0.929	259.127	3.86×10^{-8}	0.492
BPAG	0.01	0.071	524.242	1.91×10^{-8}	0.332

Table S12 The normalized values of each indicator and ToxPi scores for bisphenol compounds in the sediments of the Beijiang River Basin.

Bisphenol compounds	MC	DF	PNEC	RQ	ToxPi score
BPAP	16.90	0.214	163.993	1.03×10^{-4}	0.821
BPA	0.01	1.000	15.139	6.61×10^{-7}	0.454
BPS	1.29	1.000	352.517	3.66×10^{-6}	0.597
4-HAP	0.02	1.000	234.154	8.54×10^{-8}	0.463
BPAF	0.15	0.929	345.719	4.34×10^{-7}	0.510
4-TOP	0.01	0.214	408.035	2.45×10^{-8}	0.370
BPF	0.03	0.571	178.741	1.68×10^{-7}	0.484
BPP	0.01	0.286	302.030	3.31×10^{-8}	0.379
BPAS	0.01	0.870	259.127	3.86×10^{-8}	0.410
BPAG	0.01	0.470	524.242	1.91×10^{-8}	0.360

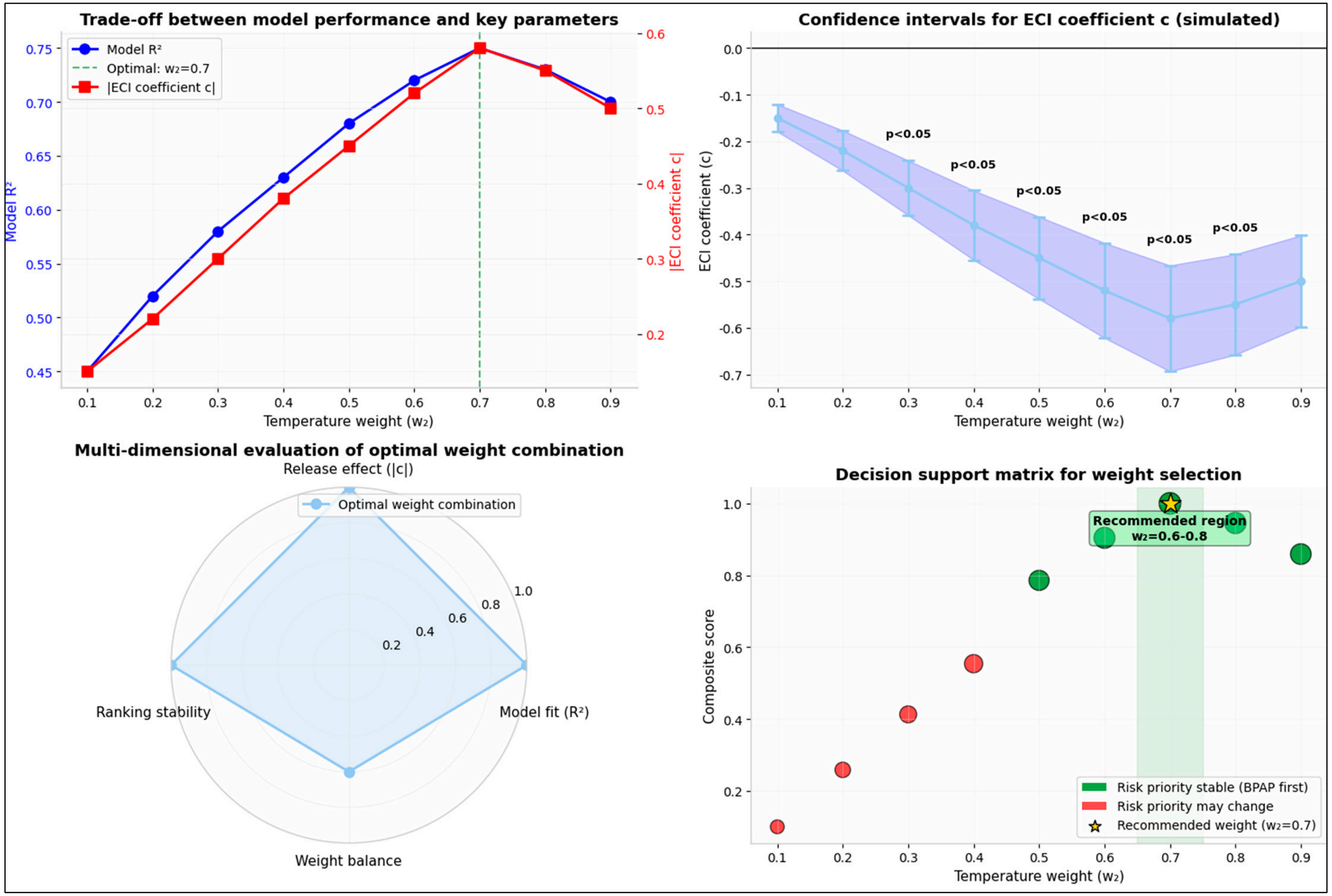

Figure S1 Determination of optimal weighting coefficients for dissolved organic carbon (*DOC*) and temperature in the

Environmental Co-occurrence Index (*ECI*) model through multi-criteria sensitivity analysis. Multi-criteria sensitivity analysis for determining the optimal weighting coefficients for dissolved organic carbon (*DOC*) and temperature(*T*) in the Environmental Co-occurrence Index (*ECI*) model. The figure illustrates the trade-off between model performance and key parameters, the multi-dimensional evaluation of weight combinations, and the decision support matrix for weight selection.

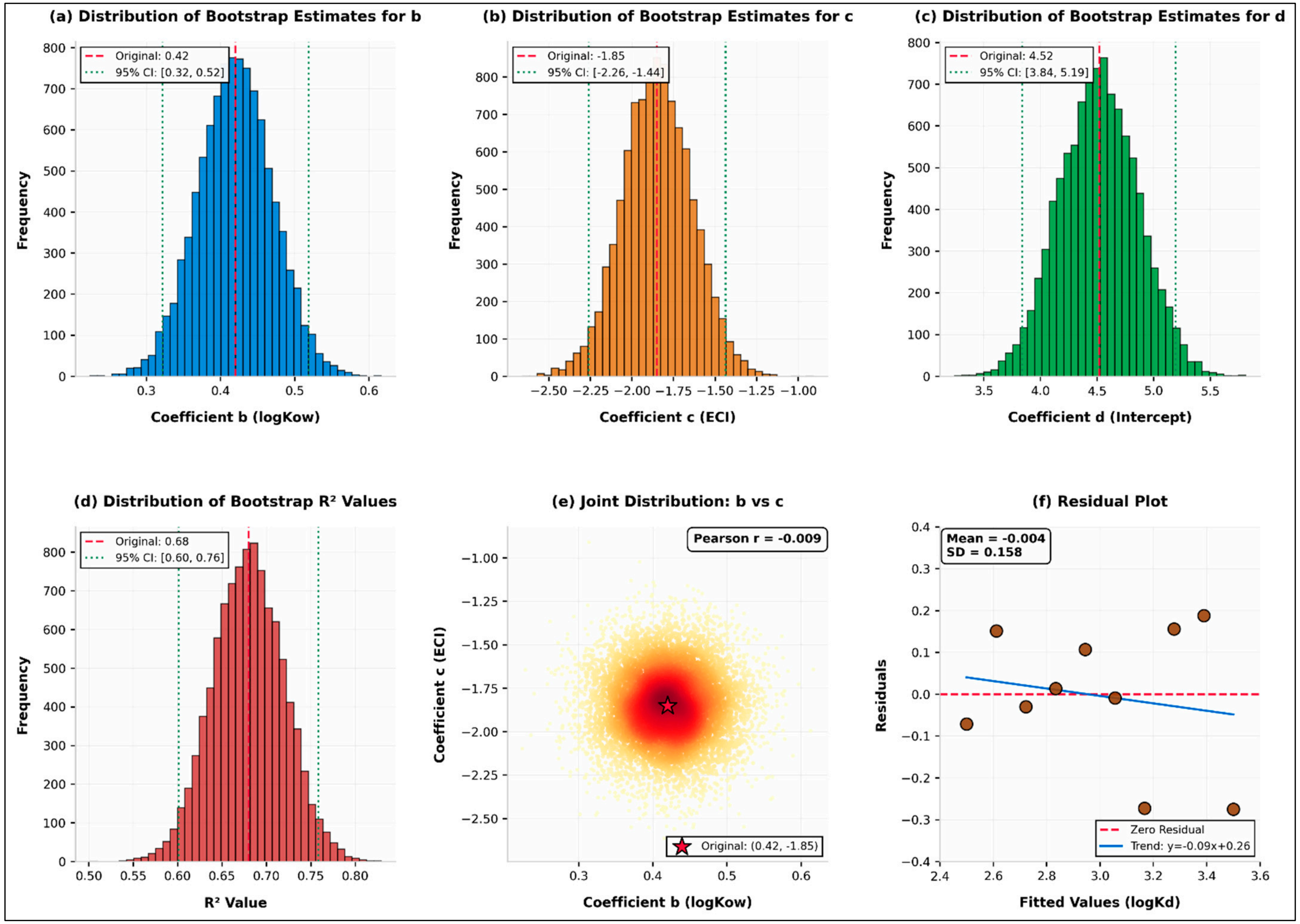

Figure S2 Bootstrap Distributions and Residual Diagnostics of the ECI Partitioning Model (with 10000 iterations). The figure assesses the stability of model parameters through bootstrap resampling and evaluates the goodness-of-fit and underlying assumptions via residual analysis.

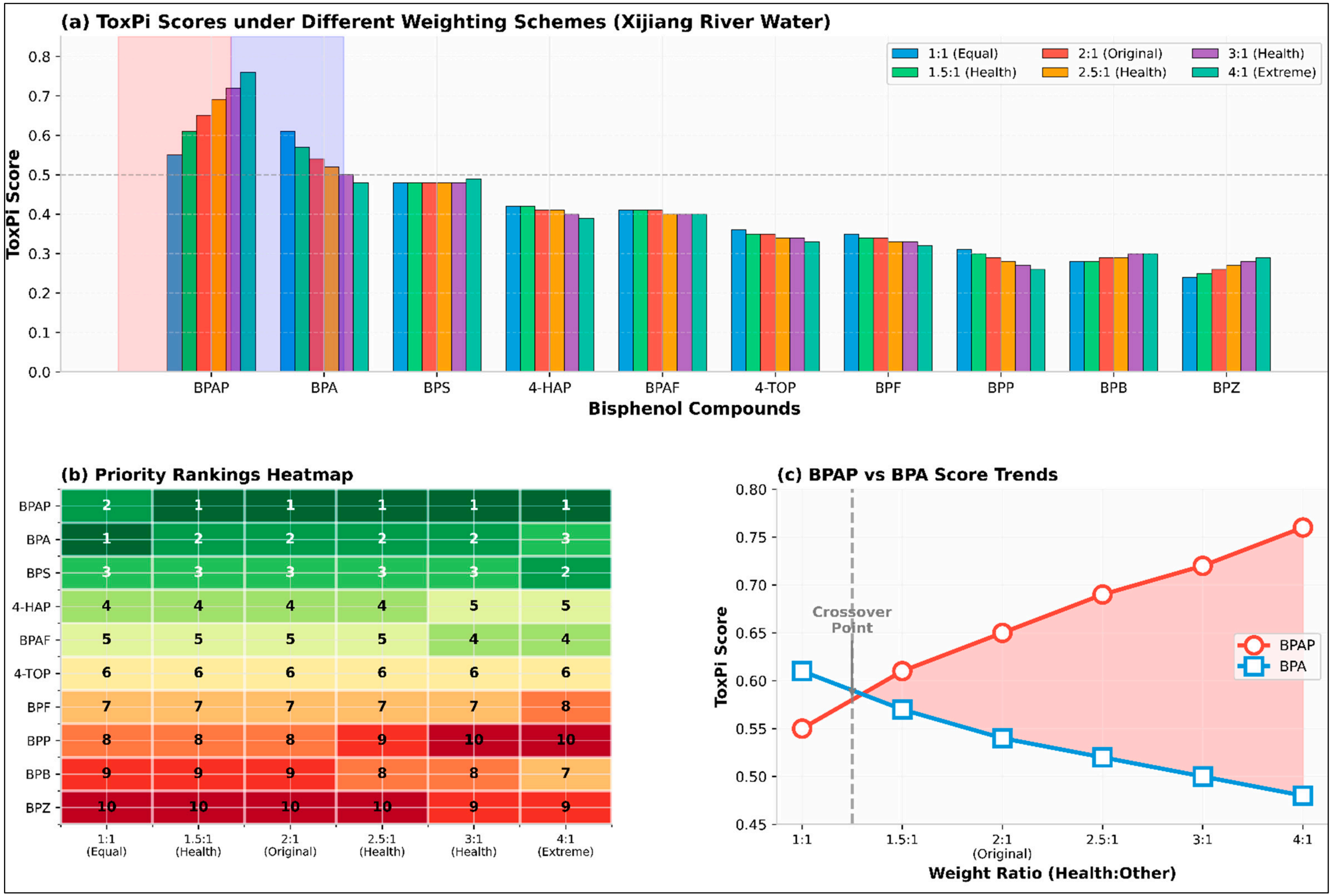

(a) ToxPi Scores under Different Weighting Schemes (Xijiang River Water)
1:1 (Equal)
1.5:1 (Health)
2:1 (Original)
2.5:1 (Health)
3:1 (Health)
4:1 (Extreme)
ToxPi Score
BPAP
BPA
BPS
4-HAP
BPAF
4-TOP
BPF
BPP
BPB
BPZ
Bisphenol Compounds
(b) Priority Rankings Heatmap
1:1 (Equal)
1.5:1 (Health)
2:1 (Original)
2.5:1 (Health)
3:1 (Health)
4:1 (Extreme)
(c) BPAP vs BPA Score Trends
Crossover Point
BPAP
BPA
ToxPi Score
Weight Ratio (Health:Other)

Figure S3 *Sensitivity analysis of the ToxPi weighting scheme for bisphenol compounds. (a) ToxPi scores under six different health-to-other indicator weight ratios (1:1 to 4:1). (b) Priority ranking heatmap across all scenarios. (c) Trend of ToxPi scores for BPAP and BPA under different weight ratios. The analysis shows that BPAP's top ranking is stable across all weighting schemes tested, providing strong evidence that the conclusion is not sensitive to the specific weight choice.*

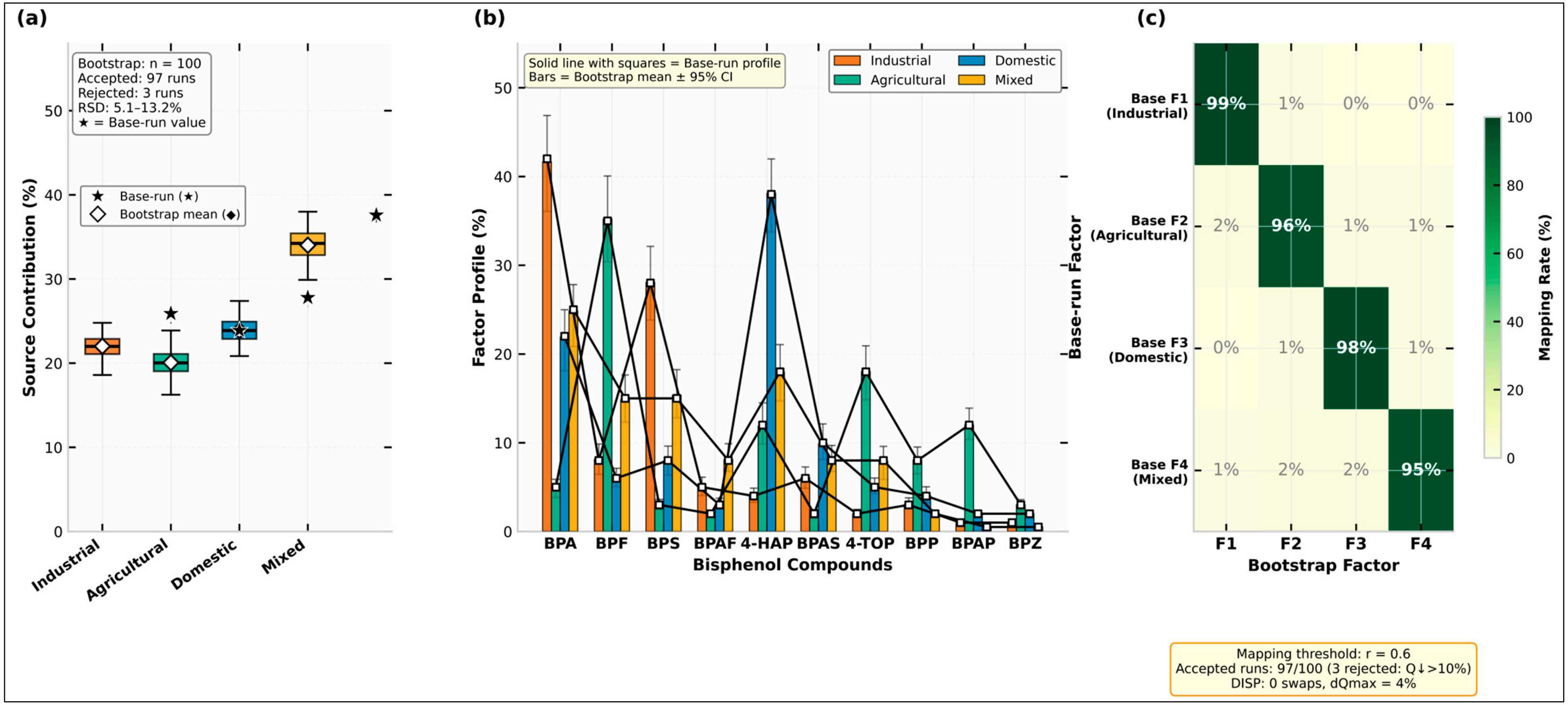

Figure S4. Bootstrap uncertainty assessment of the PMF-DMC model. (a) Distributions of source contribution percentages across 100 bootstrap runs (boxplots with 95% confidence intervals), with base-run values indicated by stars (★). Three runs were rejected due to Q-value decrease >10%, yielding 97 accepted runs. Relative standard deviations (RSD) range from 5.1% to 13.2%. (b) Factor profile stability: base-run profiles (solid lines with squares) overlaid with bootstrap means (bars) and 95% confidence intervals (error bars) for each of the four resolved sources across 10 bisphenol compounds. (c) Factor mapping reproducibility matrix (mapping threshold: Pearson r = 0.6) showing mapping rates of 94 – 99% among 97 accepted bootstrap runs. DISP analysis: zero factor swaps, dQmax = 4%. The minor off-diagonal elements (1 – 2%) for the Mixed factor reflect its inherent compositional overlap with industrial and agricultural sources.

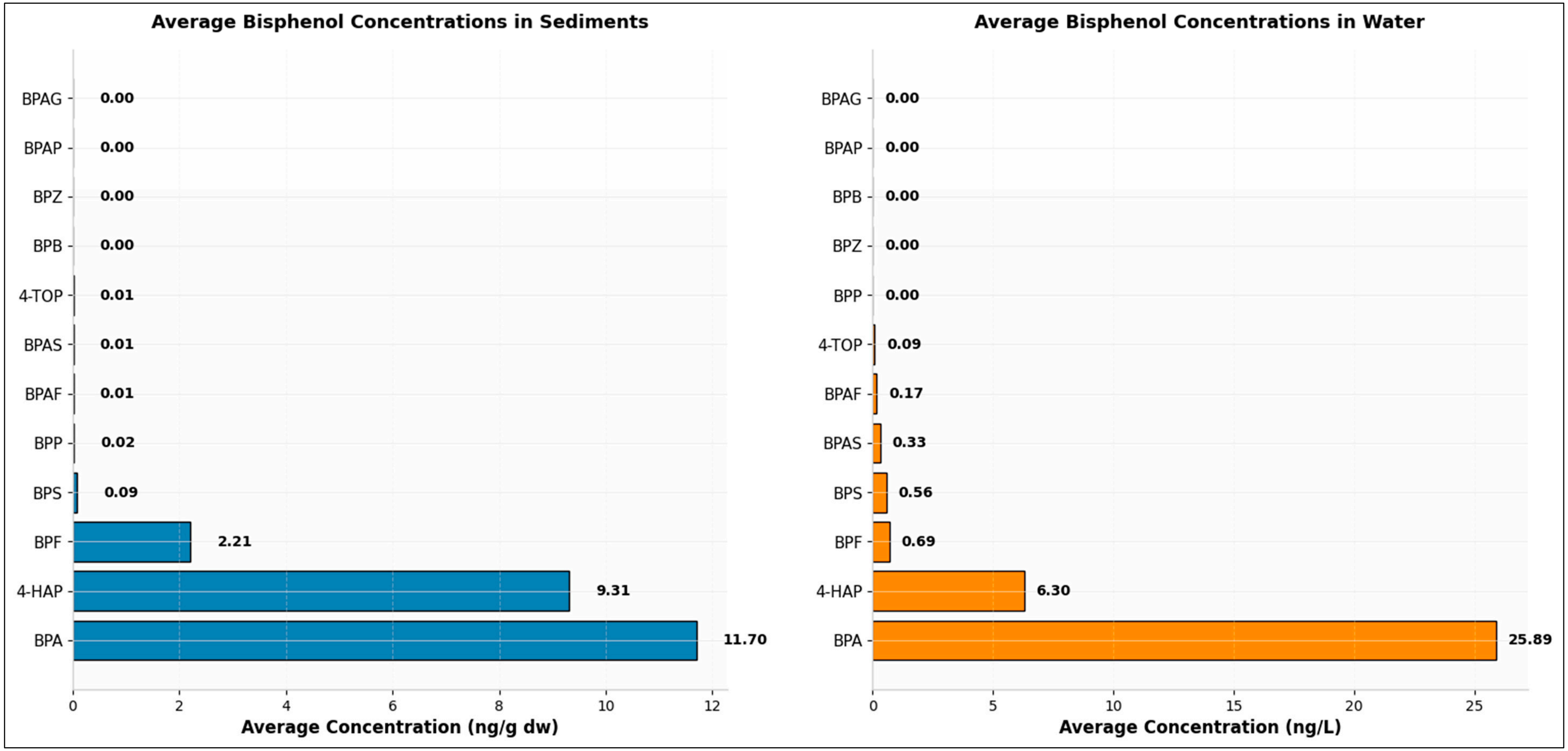

Figure S5 Comparison of average bisphenol compounds concentrations in sediment and water bodies.

Supplementary Information: Detailed Methodology for Water and Sediment Sampling, Chemical Analysis, and Model Parameter Calibration

Text S1. Comprehensive Water and Sediment Sampling Protocol

Fifteen monitoring cross-sections were strategically established within the Foshan section of the Pearl River Basin, including seven along the Xijiang River (XJ1 – XJ7) and eight along the Beijiang River (BJ1 – BJ8). The sampling network was designed to capture the full continuum of pollutant dynamics, covering basin inlets, outlets, nationally/provincially designated water quality control sections, and zones within drinking water source protection areas. Sediment samples were collected concurrently with water samples at each cross-section to ensure direct comparability between water column and sediment contamination data.

S1.2. Sampling Period and Hydrological Regime

Sampling was conducted during two characteristic hydrological periods of the Pearl River Basin to represent distinct water quality conditions:

1. Early flood season: Late June to early July, representing a period primarily driven by frontal rainfall with relatively stable baseflow.
2. Late flood season: Mid-August, representing a period dominated by typhoon-induced precipitation following major flood events.

All sampling activities were performed under clear or partly cloudy conditions. A strict protocol was followed to ensure no precipitation occurred within 48 h preceding sample collection at any site, thereby minimizing the confounding effects of transient surface runoff and ensuring that samples reflected the ambient contamination status for each period.

S1.3. In situ Measurements

Prior to sample collection, the following parameters were recorded at each site:

1. Physicochemical parameters: Water temperature, pH, and dissolved oxygen (DO).
2. Hydrological descriptors: Channel width, water depth, and flow velocity (measured using a calibrated current meter).

S1.4. Water Sample Collection

Water samples were collected following the national technical specifications for surface water environmental quality monitoring (HJ 91.2-2022). A composite sampling strategy was employed at each monitoring cross-section based on river width and water depth to ensure

representativeness of the entire water column:

1.Based on river width:

For river widths ≤ 50 m, sampling was conducted at the thalweg (main flow line).

For widths > 50 m and ≤ 100 m, samples were taken from both the left and right banks where flow was evident.

For widths > 100 m, samples were collected from the left, center, and right sections.

2.Based on water depth (at each selected lateral point):

For water depth ≤ 5 m, a sample was collected at 0.5 m below the water surface.

For depth > 5 m and ≤ 10 m, samples from 0.5 m below the surface and 0.5 m above the sediment were collected and mixed.

For depth > 10 m, a composite sample was prepared from layers at 0.5 m below the surface, mid-depth, and 0.5 m above the sediment.

Up to nine discrete sub-samples from a single cross-section were homogenized to form one integrated sample per site. This approach minimizes errors from single-point sampling and captures vertical and horizontal heterogeneity in contaminant distribution. Sampling was conducted from a boat positioned with its bow facing upstream; samples were collected from the upstream side (bow) to avoid contamination from the vessel, with the sampler opening oriented facing the current.

S1.5. Sediment Sample Collection

Sediment sampling followed the national technical specifications for environmental monitoring, specifically the Technical Specifications for Environmental Monitoring of Coastal Sea Areas—Part 1: General Rules (HJ 442.1-2020) and the Technical Guidelines on Sampling Techniques for Water Quality (HJ 494-2009). A stainless steel grab sampler was used to collect the top 5 cm of surface sediment at each designated point. To ensure sample homogeneity and remove large debris, the collected sediment was immediately passed through a 2 mm stainless-steel sieve on site. The sieved sediment was then transferred into pre-cleaned amber glass bottles.

S1.6. Sample Container Preparation and Collection

All sample containers were rinsed three times with in situ water from the sampling point prior to filling. For bisphenol compound (BP) analysis in water, samples were collected in pre-

cleaned 1 L amber glass bottles. Sediment samples were collected directly into pre-cleaned amber glass bottles after sieving.

S1.7. Sample Preservation and Transportation

1. Water samples: Immediately after collection, samples for BP analysis were acidified on site by adding 2 mL of 1 mol L^{-1} hydrochloric acid (HCl) to suppress microbial activity and prevent biodegradation of target analytes, adjusting the sample pH to below 2. Pilot experiments confirmed that this acidification protocol did not significantly affect the spiked recoveries of the target compounds.
2. Sediment samples: Following collection and sieving, sediment samples were stored in darkness at 4 °C during transport to the laboratory.

All samples were sealed immediately after preparation. Water and sediment samples were stored in darkness at 4 °C and transported to the analytical laboratory within 24 h. Upon arrival, sediment samples were promptly transferred to a -20°C freezer for long-term preservation until chemical analysis. All necessary sample processing, including extraction for sediment and preparation for water analysis, was completed within one month of collection to ensure analyte stability and data integrity. Water sample instrumental analyses were completed within one week of collection.

Text S2. Chemical Analysis and Quality Assurance/Quality Control (QA/QC)

S2.1. Target Compounds

Target compounds included 12 bisphenol compounds (BPs, Table S4): Bisphenol A (BPA), Bisphenol B (BPB), Bisphenol F (BPF), Bisphenol P (BPP), Bisphenol S (BPS), Bisphenol Z (BPZ), Bisphenol AF (BPAF), Bisphenol AP (BPAP), 4'-Hydroxyacetophenone (4-HAP), Bisphenol A monosulfate sodium salt (BPAS), Bisphenol A glucuronide (BPAG), and 4-tert-Octylphenol (4-TOP). Due to the lack of key toxicity parameters (e.g., Reference Dose, RfD) for BPAS and BPAG in water, and the non-detection of BPB and BPZ in sediments, subsequent risk assessment and ToxPi analysis focused on the remaining 10 BPs with complete toxicity data.

S2.2. Sample Preparation and Instrumental Analysis

Water samples: A 1 L water sample was filtered through a 0.7 μm glass fiber filter to

remove suspended particulate matter. The filtrate pH was adjusted to 5 – 7 using dilute HCl or NaOH solution. After adding appropriate internal standards and 5 g of NaCl, liquid–liquid extraction was performed using 50 mL of acetonitrile (vortexed for 5 min, then allowed to phase separate). The organic phase was collected, and the extraction was repeated once. The combined extracts were dehydrated with anhydrous Na_2SO_4, concentrated to near-dryness under a gentle nitrogen stream, reconstituted to 0.1 mL with acetonitrile, and finally filtered through a 0.22 μm organic-phase filter before instrumental analysis.

Sediment samples: Approximately 2.0 g (dry weight) of sediment was accurately weighed into an extraction cell, and an appropriate amount of internal standard (BPA-d_{16}) was added. Extraction was performed using an accelerated solvent extractor (ASE 350, Dionex, Sunnyvale, CA, USA) with a solvent mixture of dichloromethane:acetone (1:1, v/v) at 100 °C and 1500 psi, with a static extraction time of 5 min for two cycles. The extract was concentrated under reduced pressure using a rotary evaporator, subsequently loaded onto a composite solid-phase extraction cartridge packed with silica gel and Florisil for matrix cleanup, and eluted with a 1:1 (v/v) mixture of n-hexane and dichloromethane. The eluate was concentrated under nitrogen, solvent-exchanged to acetonitrile, adjusted to a final volume of 0.1 mL, filtered through a 0.22 μm filter, and then analyzed.

S2.3. Instrumental Analysis

Sample analysis was conducted using a liquid chromatography–tandem mass spectrometry system (LC-MS/MS) comprising an LC-30AD UHPLC (Shimadzu, Kyoto, Japan) coupled to a QTRAP® 5500 triple quadrupole mass spectrometer (AB Sciex, Framingham, MA, USA), equipped with an electrospray ionization (ESI) source operating in negative ion mode. Chromatographic separation was performed on a Waters ACQUITY UPLC® HSS C_{18} column (2.1 × 100 mm, 1.8 μm, Waters Corporation, Milford, MA, USA) maintained at 40 °C. The mobile phase consisted of (A) 2 mmol L^{-1} ammonium acetate in water and (B) acetonitrile, delivered under the following gradient program: 0 – 0.5 min, 10% B; 0.5 – 4.0 min, linear increase from 10% to 60% B; 4.0 – 9.0 min, linear increase from 60% to 80% B; 9.0 – 9.1 min, rapid re-equilibration from 80% to 10% B; and 9.1 – 12.0 min, isocratic hold at 10% B. The flow rate was 300 μL min^{-1}, and the injection volume was 2 μL. The ESI source was operated at 550 °C with a spray voltage of –4500 V. Data acquisition was performed in multiple

reaction monitoring (MRM) mode using Analyst® software (version 1.7, AB Sciex, Framingham, MA, USA.

S2.4. Quality Assurance and Quality Control (QA/QC)

A rigorous QA/QC program was implemented to ensure data integrity. During instrumental analysis, a full suite of QC samples, including laboratory blanks, matrix blanks, matrix spikes (MSs), and matrix spike duplicates (MSDs), was analyzed after every 20 environmental samples. Quantification of BPs was performed using the internal standard method. Method detection limits (MDLs), determined at a signal-to-noise ratio (S/N) of 3, were 0.05 ng L^{-1} for water and 0.025 ng g^{-1} dry weight (dw) for sediments. Instrumental stability was monitored by analyzing a calibration verification standard every ten samples; response deviations for all target compounds remained within ±20%. No target BPs were detected in any blank samples (i.e., concentrations were below the MDL).

To provide context for the recovery results, the following details are now included: For the water matrix, MS and MSD samples were prepared by fortifying 1 L of a pre-screened (non-detect) water sample with the target BP standard mixture at a total spiked mass of 50 ng (equivalent to a nominal concentration of 50 ng/L). This spike level was selected to be within the same order of magnitude as the total BP concentrations measured in our field samples (e.g., the average total BP concentration in the study area was 71.8 ng/L for BPA alone), ensuring that the reported recovery accurately reflects method performance within the environmentally relevant concentration range. The mean recovery of the 12 BPs in matrix-spiked samples was 93.1 ± 7.93% (n = 6 per matrix). Surrogate recoveries in field samples ranged from 56.3 ± 12.1% to 113 ± 22.3% (n = 3 – 5 per compound), all satisfying established QA/QC acceptance criteria (40 – 130%).

Text S3. Calibration of Dual-Media Weight (*w*) for the PMF-DMC Model

S3.1. Objective

The dual-media weight (*w*) is a critical parameter in the Positive Matrix Factorization–Dual-Media Coupling (PMF-DMC) model. It quantifies the average mass distribution of target contaminants between the water phase and sediment phase across the study basin, thereby determining the relative contribution of each medium to the final integrated source

apportionment results. This section details the data-driven calibration process for *w*.

S3.2. Methodology

The calibration was based on field-measured solid−water distribution coefficients (log K_d) of target compounds. The procedure was as follows:

1. Pollutant selection: Seven bisphenol compounds and derivatives with valid log K_d values (from either the early or late flood season) were selected: BPA, BPF, BPS, BPAF, 4-HAP, BPAS, and 4-TOP.
2. Average log K_d calculation: For each pollutant, the arithmetic mean of its log K_d values from the early and late flood seasons was calculated.
3. Water-phase fraction calculation: The mass fraction residing in the water phase (F_{water}) for each pollutant was calculated using the following formula: F_{water} = 1 / (1 + 10^ (log$K_{d,\ avg}$).
4. Model weight determination: The dual-media weight for the water phase (W_{water}) was defined as the arithmetic mean of the F_{water} values from the seven pollutants. The weight for the sediment phase ($W_{sediment}$) was consequently derived as $W_{sediment} = 1 - W_{water}$.

S3.3. Results

The calculated water phase fractions (F_{water}) for the seven pollutants were:

BPA	BPF	BPS	BPAF	4-HAP	BPAS	4-TOP
0.874	0.617	0.922	0.851	0.458	0.992	0.879

The average F_{water}, and thus the calibrated W_{water}, was 0.73 (rounded from 0.727). Consequently, $W_{sediment}$ was 0.27.
